# Naringenin Nano-Delivery Systems and Their Therapeutic Applications

**DOI:** 10.3390/pharmaceutics13020291

**Published:** 2021-02-23

**Authors:** Mohammed Bhia, Mahzad Motallebi, Banafshe Abadi, Atefeh Zarepour, Miguel Pereira-Silva, Farinaz Saremnejad, Ana Cláudia Santos, Ali Zarrabi, Ana Melero, Seid Mahdi Jafari, Mehdi Shakibaei

**Affiliations:** 1 Student Research Committee, School of Pharmacy, Shahid Beheshti University of Medical Sciences, Tehran 1996835113, Iran; mohamadbahia1966@gmail.com; 2 Nanomedicine Research Association (NRA), Universal Scientific Education and Research Network (USERN), Tehran 7616911319, Iran; 3 Department of Biology, Yadegar-e-Imam Khomeini (RAH) Shahr-e-Rey Branch, Islamic Azad University, Tehran 1815163111, Iran; mahzadmotallebi79@gmail.com; 4 Pharmaceutics Research Center, Institute of Neuropharmacology, Kerman University of Medical Sciences, Kerman 7616911319, Iran; banafshe.abadi@yahoo.com; 5 Brain Cancer Research Core (BCRC), Universal Scientific Education and Research Network (USERN), Tehran 1419733151, Iran; 6 Department of Biotechnology, Faculty of Biological Science and Technology, University of Isfahan, Isfahan 8174673441, Iran; atefeh.zarepour@gmail.com; 7 Department of Pharmaceutical Technology, Faculty of Pharmacy, University of Coimbra, 3000-548 Coimbra, Portugal; miguelsp_20@hotmail.com (M.P.-S.); acsantos@ff.uc.pt (A.C.S.); 8 REQUIMTE/LAQV, Group of Pharmaceutical Technology, Faculty of Pharmacy, University of Coimbra, 3000-548 Coimbra, Portugal; 9 Department of Food Science and Technology, Ferdowsi University of Mashhad, Mashhad 9177948974, Iran; fsaramnejad@gmail.com; 10 Sabanci University Nanotechnology Research and Application Center (SUNUM), Tuzla, Istanbul 34956, Turkey; alizarrabi@sabanciuniv.edu; 11 Center of Excellence for Functional Surfaces and Interfaces (EFSUN), Faculty of Engineering and Natural Sciences, Sabanci University, Tuzla, Istanbul 34956, Turkey; 12 Department of Pharmacy and Pharmaceutical Technology and Parasitology, Faculty of Pharmacy, University of Valencia, Avda. Vincent Andrés Estellés s/n, 46100 Burjassot, Spain; 13 Department of Food Materials and Process Design Engineering, Gorgan University of Agricultural Sciences and Natural Resources, Gorgan 4918943464, Iran; 14 Musculoskeletal Research Group and Tumor Biology, Chair of Vegetative Anatomy, Institute of Anatomy, Faculty of Medicine, Ludwig-Maximilian-University Munich, 80336 Munich, Germany

**Keywords:** naringenin, nutraceuticals, antioxidants, natural products, nanoparticles, drug delivery, nanomedicine, bioavailability, flavonoid

## Abstract

Naringenin (NRG) is a polyphenolic phytochemical belonging to the class of flavanones and is widely distributed in citrus fruits and some other fruits such as bergamot, tomatoes, cocoa, and cherries. NRG presents several interesting pharmacological properties, such as anti-cancer, anti-oxidant, and anti-inflammatory activities. However, the therapeutic potential of NRG is hampered due to its hydrophobic nature, which leads to poor bioavailability. Here, we review a wide range of nanocarriers that have been used as delivery systems for NRG, including polymeric nanoparticles, micelles, liposomes, solid lipid nanoparticles (SLNs), nanostructured lipid carriers (NLCs), nanosuspensions, and nanoemulsions. These nanomedicine formulations of NRG have been applied as a potential treatment for several diseases, using a wide range of in vitro, ex vivo, and in vivo models and different routes of administration. From this review, it can be concluded that NRG is a potential therapeutic option for the treatment of various diseases such as cancer, neurological disorders, liver diseases, ocular disorders, inflammatory diseases, skin diseases, and diabetes when formulated in the appropriate nanocarriers.

## 1. Introduction

Flavonoids, as polyphenol components with more than 4000 varieties, are among the most attractive classes of bioactive materials found in fruits, vegetables, medical herbs, and beverages. They show interesting physiological effects, such as free radical scavenging, metal chelation, induction of cell apoptosis, and prevention of cell proliferation. They are categorized into different classes, including flavones, isoflavones, flavonols, flavanones, and anthocyanidins. One of the most important and interesting flavonoids from the pharmaceutical point of view is naringenin (NRG) [1,2,3,4,5].

**Figure 1:**
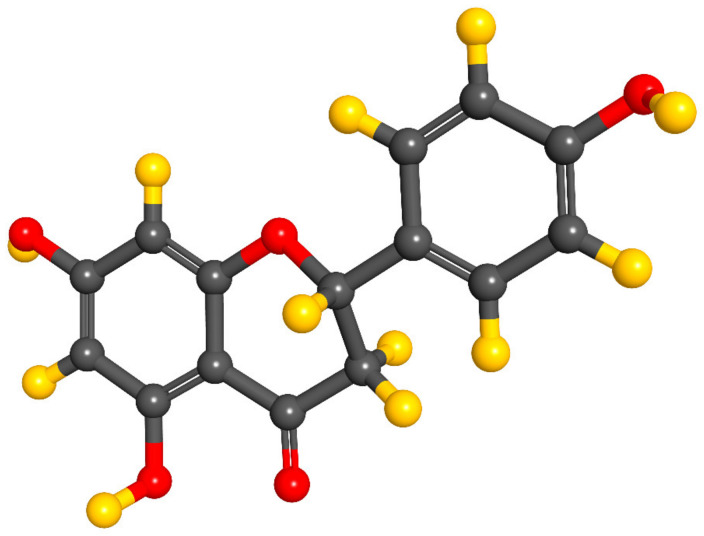
Chemical structure of naringenin. Gray spheres: carbon atoms; yellow spheres: hydrogen atoms; red spheres: oxygen atoms.

NRG is a type of flavanone (Figure 1), discovered in 1907 by Power and Tutin, as chalcone. The chemical nomenclature of this hydrophobic molecule is 2,3-dihydro-5,7-dihydroxy-2-(4-hydroxyphenyl) 4H-1-benzopyran-4-one. NRG is soluble in organic solvents such as alcohol and can be found in both the aglycol form, NRG, or in its glycosidic form. It is naturally present in citrus fruits such as oranges, lemons, grapes, tangerine, grapefruits, and other fruits such as bergamot, tomatoes, cocoa, and cherries [6,7,8,9,10,11,12]. According to PubChem, NRG is a solid with a melting point of 83 °C. It presents a molecular weight of 580.5 g/mol, a logP of -0.44 and a solubility of 1 mg/mL at 40 °C in water [13].

NRG presents several important interesting properties from the pharmacological point of view, such as anti-cancer properties, insulin-like actions for diabetes treatment, antioxidant and anti-inflammatory effects in hypertension conditions, and several other features such as anti-mutagenic, anti-proliferation, anti-fibrogenic, anti-atherogenic, antibacterial, anti-atherosclerotic, neuroprotective, antidiabetic, immunomodulatory, hepatoprotective, cardioprotective, and oxidative stress reversing properties [14,15,16,17,18,19,20,21,22,23,24,25]. Indeed, NRG could scavenge the hydroxyl and superoxide radicals, thus reducing their effect on biomolecules (such as proteins, lipids, and DNAs) in inflammatory or oxidation conditions. It can also induce cytotoxic and apoptotic effects and prevent cell proliferation (even at low concentrations) in different types of cancer cells [26,27,28,29,30,31,32,33]. It can down-regulate the pro-inflammatory mediators, such as the intercellular adhesion molecule-1 (ICAM-1), cyclo-oxygenase-2 (COX-2), tumor necrosis factor-α (TNF-α), and interleukin-6 (IL-6) for controlling diabetes and preventing obesity via reduction of the total amount of cholesterol and triglyceride contents in plasma and liver [34,35,36,37]. It also shows antibacterial activity against different bacterial strains [19,38,39,40]. 

Unfortunately, the in vivo bioavailability of NRG is very low, as a result of its hydrophobic nature, thus limiting its practical use. It shows a short half-life and is rapidly converted to its crystalline form, which has a low absorption through the digestive system [41,42,43,44]. These limitations in the use of NRG have led to several efforts to improve its bioavailability. One of the most promising candidates is the use of delivery systems at the nano-scale range. Nanomaterials present several advantages for drug delivery, as they can protect the drug during storage and after administration. They also allow controlled release and targeting to specific organs or tissues, improving their performance via increasing their bioavailability and reducing their undesirable side effects. The nanosize of these components and their engineered surface helps them pass through the vessels to reach a specific organ [45,46,47,48,49,50,51,52,53,54,55,56,57]. Thus, nanocarriers have been extensively used for the encapsulation of different phytochemicals, including NRG or naringin which is its glycosidic form [58,59,60,61,62,63,64,65].

So far, several types of nanocarriers have been fabricated for NRG delivery that enhance its solubility in water, biocompatibility, bioavailability, and therapeutic efficiency, which is also translated into dose reductions. Among these, polymeric nanocarriers (natural and synthetic) can be cited: lipid-based nanocarriers, polymeric nanoparticles, dendrimers, hydrogels, micelles, protein-based nanoparticles, carbon-based nanocarriers, nanotransfersomes, nanoemulsions, nanocomposites, metal oxide nanoparticles, etc. These particles can encapsulate NRG inside their structures and release it in a controllable manner [66,67,68,69,70,71,72,73,74,75,76,77,78,79,80,81,82,83,84]. 

Based on these features, our aim is to review the literature in the search of different reported NRG-loaded nanoformulations, formulated to improve NRG delivery with different therapeutic objectives. First, we discuss about the various limitations of NRG delivery. Then, we describe some of the nanoformulations used for delivering NRG and its therapeutic applications to treat different types of diseases. Finally, we conclude with a future perspective comment of this active herbal ingredient.

## 2. Methods

The literature search to prepare this work was carried out in June 2020 on the Internet using the search engines “PubMed”, “Scopus” and “Science Direct”. The keywords used were: “naringenin” AND “Nano*” OR “liposom*” OR “micell*” OR “drug delivery”. 

From the three search engines, given that the purpose of this work has been to provide an overview of the current knowledge about naringenin applications in nanocarriers and it is a new research area, the type of articles selected preferably were original scientific articles. A total number of 177 articles were used in this review. To offer the most up-to-date information possible, the years included in the search period were between 2005 and 2020. The language of the studies analysed was English. The articles were ordered, firstly, according to “best match” and later according to “most recent” to adequately draw conclusions. Both in vitro and in vivo studies were considered. 

The information was first organized according to the nanocarrier used to improve naringenin delivery drawbacks. Secondly, the information was organized according to the treated disease.

## 3. NRG Bioavailability and Delivery Challenges

**Figure 2:**
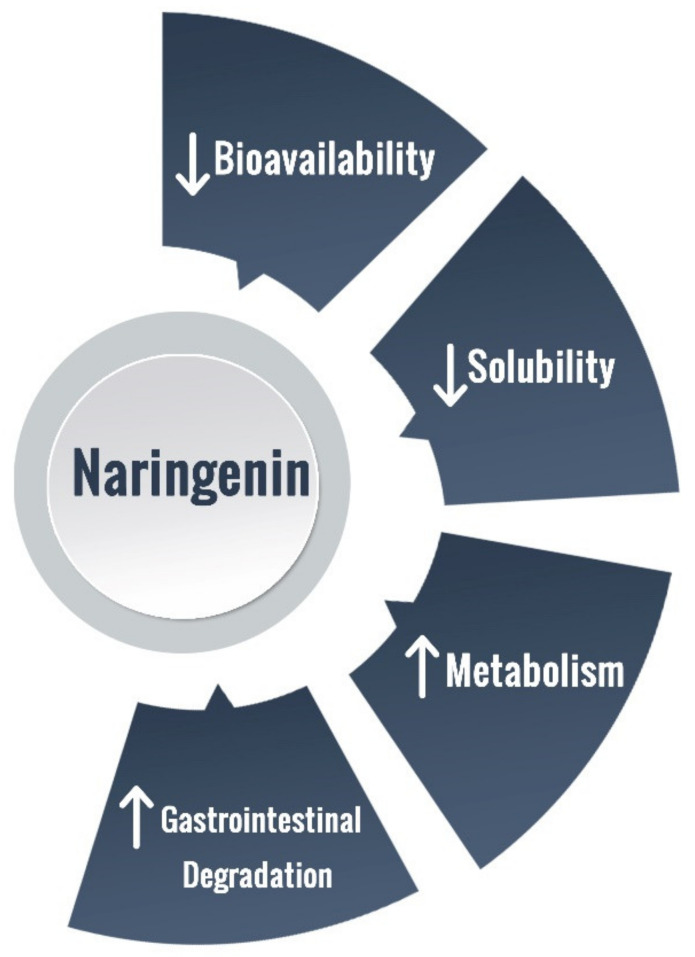
Delivery limitations of naringenin.

NRG is present in a wide range of foods, being characteristic of a normal diet intake. However, NRG’s biological functions are hampered due to its hydrophobic nature, evidencing a poor aqueous solubility (ca. 475 mg/L) [11], as well as to an extensive gastrointestinal degradation, liver first-pass metabolism, and limited membrane transportation, which overall contribute to a reduced oral bioavailability (Figure 2) [2,11,85,86]. NRG absorption encompasses passive diffusion and active transport [11]. Yang Bai et al., 2020, studied the pharmacokinetics and metabolism of naringin and naringenin, after oral and intravenous administration of naringin in humans. The authors found that the plasma naringin concentration increased to its maximum at about 2 h (Tmax, 2.09 ± 1.15 h) and decreased to 50% of Cmax at about 3 h (t 1/2, 2.69 ± 1.77 h). Naringenin showed a considerable lag-time in human plasma (5.74 ± 2.38 h) after oral administration, due to intestinal metabolism, and the naringenin concentrations increased to Cmax at about 3.62 ± 3.19 h. The authors also studied the pharmacokinetic parameters of naringin and naringenin in food-effect trials. Tmax, Cmax, and AUC, were insignificantly altered by a high-fat diet. Furthermore, there were significant differences between the pharmacokinetic parameters of males and females humans, being some pharmacokinetic parameters of females significantly higher compared to males [87]. Several reports point toward NRG rapid in vivo metabolization, mainly through conjugation into sulphates and glucuronides, the major plasma circulating NRG derivatives [86]. Twelve metabolites of naringin and naringenin were identified in human liver and kidney microsomes: naringin, hesperidin, hesperetin, naringenin-*O*-glucoside, naringenin-*O*-glucuronide, neoeriocitrin, rhoifolin, naringenin, eriodictyol, apigenin, and 5, 7-dihydroxychromone. Yang Bai et al., 2020, demonstrated that CYP2C9 produced six of the twelve metabolites; CYP2C19, four metabolites; CYP2D6, three metabolites; CYP3A4/5, two metabolites; and CYP1A2, one metabolite [87].

In addition, the susceptibility to oxidative modifications and degradative potential in aqueous solutions can negatively impact the ultimate pharmacological properties and reduce the shelf life of NRG-containing formulations [82]. The additional metabolization of flavanones in the colon can yield small absorbable phenolic compounds, thus achieving considerably higher plasmatic concentrations when compared to native flavanones [88,89]. The poor bioavailability of NRG could be increased by replacing the natural glycoside with the respective aglycones, thus leveraging NRG absorption [85].

To overcome the restraints associated with the low bioavilability, several NRG-loaded nanocarriers, bearing distinct physicochemical composition and biological attributes, have been developed to improve NRG stability, solubility, barrier crossing, and bioavailability at target sites, with already described applications and encouraging results, achieved for a plethora of conditions or diseases [6,11].

## 4. NRG Nano-Scaled Delivery Systems

### 4.1. Polymeric Nanoparticles

Since the 1980s, various polymeric nanocarriers such as polymeric micelles, dendrimers, nanogels, nanocapsules, and other nanovesicles have been applied as drug delivery systems to transport different drugs to targeted organs [90,91]. Polymeric nanoparticles are considered nanocarriers made of biocompatible and biodegradable polymers. Depending on their preparation method, these particles are mostly presented in the form of nanocapsules or nanospheres [92,93]. Nanospheres are matrix-type delivery systems based on uniform drug encapsulation within the polymer chains, whereas, in the nanocapsule system, the drug is placed at the center and is surrounded by a polymeric membrane [93]. Polymeric nanoparticles could deliver various kinds of molecules, such as proteins, plasmids, antisense DNA, and drugs with low molecular weight. In this kind of delivery system, the particle size, surface charge distribution, and hydrophobicity and hydrophilicity of the polymeric nanoparticles are the properties that determine their ability to reach the interested organ [91,92]. The application of polymeric nanoparticles in drug delivery improves their safety profile, improves the biocompatibility and bioavailability, enhances drug permeability and stability, provides protection against hydrolytic enzymes, and improves treatment effectiveness, compared to conventional therapeutic methods [92,93,94,95,96,97].

A number of studies employed polymeric nanoparticles as NRG delivery vehicles [66]. A study in 2016 investigated the in vivo toxicological properties of poly (vinyl pyrrolidone) (PVP)-coated NRG nanoparticles. PVP is a nontoxic, water-soluble polymer used as a delivery system to enhance the solubility and bioavailability of poor water-soluble agents and reduce their complement activities. In this research, the PVP-coated NRG was synthesized by the nanoprecipitation method, which obtained a particle size of 110 nm. Particles were characterized by X-ray diffraction (XRD), field emission scanning electron microscopy (FE-SEM), Fourier transform infrared (FTIR) spectra, and energy dispersive X-ray spectroscopy (EDX). In vivo toxicological evaluations at different doses in male Sprague–Dawley (SD) rats were done comparing the particles with silver nanoparticles (AgNPs). Analysis of hepatotoxicity markers, hematological parameters, antioxidant enzymes in liver, kidney, and heart, and mRNA expressions of nuclear factor kappa-light-chain-enhancer of activated B-cells (NF-κB), TNF-α, and IL-6, which are involved in inflammatory cascades, showed no significant difference in PVP-coated NRG nanoparticles in comparison with the control group, whereas in AgNPs treated rats the amount of all these parameters were higher than the control group. The results proved that the highly safe nanoparticles could be employed as a suitable NRG delivery system in the field of biomedicine [44].

Micelles are polymeric nanocarriers made of amphiphilic polymeric molecules that can be applied to encapsulate hydrophobic drugs in their core [90]. The delivery of NRG via micelles has been studied by several research groups. In 2015, the impact of encapsulating NRG in a mixed micelle formation of Pluronic F127 and Tween 80 on its oral bioavailability was evaluated in male SD rats [98]. Pluronic F127 (Poloxamer 407) is a non-ionic surfactant, and its micellar properties are highly soluble and suitable for drug delivery [99]. Tween 80 (polyoxyethylene sorbitan monooleate) is also a non-ionic surfactant. Due to its low toxicity rate, low cost, and high solubility it has been widely used for drug delivery [100]. In this study, the NRG-loaded mixed micelles were made by a thin-film hydration technique, with a ratio of NRG: Pluronic F127: Tween 80 of 1:10:0.2 (*w/w/w*). The in vivo investigation of their pharmacokinetic parameters showed a significant enhancement in NRG solubility (by 27-fold), its bioavailability (up to 26.9% following oral administration), and intestinal permeability (1.7-fold) compared to free NRG administration. Hence, it could be concluded that NRG-loaded micelles are more effective than oral administration of free NRG [98].

**Figure 3:**
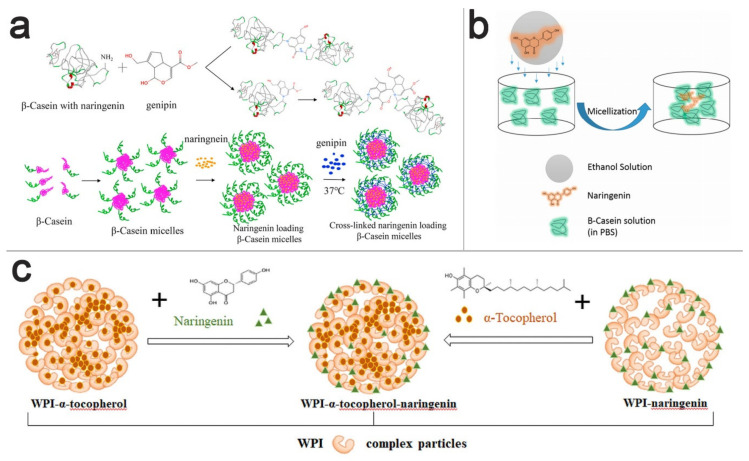
Different nanoparticles for the delivery of naringenin. (**a**) Genipin-crosslinked ß-casein micelles. Reproduced with permission from [101], Elsevier, 2020. (**b**) Nanosized naringenin-(ß-casein) complexes. Reproduced with permission [103]. (**c**) Whey protein isolate (WPI) as nanocarriers for naringenin. Reproduced with permission from [104], Elsevier, 2020.

Another study on micelles as NRG delivery system is done in 2020 by Mo Li et al., in which they investigated NRG-loaded genipin-crosslinked ß-casein micelles (G-CNMs) [101]. Caseins are proteins obtained from cow milk, which, due to their inexpensive, stable, bioavailable, and biodegradable characteristics, have gained special attention as carriers for bioactive compounds [102]. In the research conducted by Mo Li et al., ß-casein micelles were prepared, loaded with NRG, and further crosslinked with genipin (Figure 3a) (G-CNMs). According to sodium dodecyl sulfate-polyacrylamide gel electrophoresis (SDS-PAGE) and gel permeation chromatography-multi angle light scattering (GPC-MALLS) assays, the micellar crosslinking rate was genipin concentration-dependent. At the ß-CN/genipin molar ratio of 1:20, the average hydrodynamic radius of G-CNMs calculated by light scattering was 9.35 nm. As a result of genipin crosslinking, the conformation of G-CNMs had a significant transition from random coil into ß-sheet. The results indicated that, when the ß-CN/genipin molar ratio was 1:15 or 1:20, G-CNMs dominated the instability of micelles and was caused by an acidic pH (pH = 2). In contrast, there were no changes in stability at a high amount of NaCl. NRG was released slowly from all kinds of G-CNMs in phosphate-buffered saline (pH = 7.4). However, after increasing the amount of genipin, the NRG release was enhanced, which means that the stability of CNMs and NRG controlled release is influenced by the degree of genipin crosslinking. Taken together, ß-CN could be a suitable nanocarrier for hydrophobic drugs such as NRG with the attention to genipin as an important agent for protein particles [101].

The binding of NRG with ß-casein has been investigated in another study using fluorescence and UV-Vis absorption spectroscopy. The nanosized NRG-(ß-casein) complexes were prepared with various volumes of NRG at room temperature (Figure 3b). The findings indicated that NRG suppressed the intrinsic fluorescence of Trp 143 residue, and according to the thermodynamic analysis, the interaction between NRG and ß-casein is spontaneous with respect to the importance of hydrophobic interactions, *Van Der Waals* forces, and hydrogen bonds [103].

NRG-loaded monomethoxy poly (ethylene glycol)-poly(Ɛ-caprolactone) (MPEG-PCL) nanoparticles were also prepared to investigate the effects of their formulation into buccal tablets in NRG solubility and in the treatment of oral inflammation [105]. MPEG-PCL is an amphiphilic copolymer that has attracted interest as a drug carrier thanks to its biodegradability, biocompatibility, and low toxicity profile. In this research, NRG-loaded nanoparticles were made using a solvent evaporation method. To prepare buccal tablets, NRG nanoparticles were freeze-dried into stable and re-soluble nanoparticle lyophilized powder. The physicochemical properties of NRG nanoparticles such as particle size, TEM, and in vitro release were investigated. The results showed a small particle size and high drug loading capacity. To check the in vitro release of buccal tablets, the US Pharmacopeia 1 method was used. Buccal tablets containing NRG nanoparticles produced a faster and complete release compared to the control group over 12 h. These findings are promising for the application of NRG to deliver anti-inflammatory drugs in oral disease [105]. 

The use of native and preheated ß-lactoglobulin (ß-lg), an abundant whey protein, as a carrier for NRG, compared to its glycosylated form (naringin), was studied by Shpigelman et al. The prepared NRG solution was added to a ß-lg solution in pH = 7 phosphate buffer. UV spectrophotometry and intrinsic fluorescence methods showed NRG binding with both preheated and native ß-lg, with no effects on the binding stoichiometry (2:1 ß-lg to NRG). No binding between the glycosylated form naringin and ß-lg was seen, which could be a result of its larger size and more hydrophilic nature. The size of NRG-loaded nanoparticles, estimated by dynamic light scattering (DLS), showed a small increase compared to the pure protein. NRG-ß-gl complexes showed increased solubility (up to 3 times) with no crystal formation and acceptable reconstitution after freeze-drying. To conclude, the solution obtained was clear, thus suitable to enrich clear beverages with hydrophobic compounds such as NRG [43]. Among various food proteins used as bioactive carriers, whey protein isolate (WPI) is one of the most widely used. In a study conducted by Yin et al. in 2020, the antioxidant feature, stability, and bioaccessibility of hydrophobic α-tocopherol and amphiphilic NRG loaded into WPI nanoparticles were investigated (Figure 3c). The findings demonstrated that the antioxidant activity of protein-NRG particles was increased, whereas there was no effect of WPI on NRG storage and digestive, but the storage stability of α-tocopherol was improved. The drug bioavailability showed improvement by adding NRG, plus the fact that the oxidation of NRG and α-tocopherol did not damage proteins. In conclusion, WPI could make an excellent nanocarrier to encapsulate and transfer different compounds [104].

Chitosan and dextran sulfate has also been used to encapsulate NRG with the aim to improve its therapeutic properties. The chitosan dextran sulfate NRG nanoparticles were spherical in shape, and their cytotoxic effects on breast cancer cell line (MCF-7) were evaluated by 3-(4,5-dimethylthiazol-2-yl)-2,5-diphenyl-2H-tetrazolium bromide (MTT) assay after 24 h incubation. The results indicated considerable cytotoxic activity of chitosan-dextran sulfate-NRG in addition to the controlled but rapid release of 80% of free NRG over 36 h. Hence, it seems that chitosan–dextran–sulfate–NRG nanocarrier is a suitable delivery system for NRG and other hydrophobic drugs [106].

### 4.2. Lipid-Based Nanoparticles

During the past two decades, lipid-based drug delivery has been at the center of attention to tackle conventional formulations’ limitations, especially for drugs that are not properly soluble in water [107,108,109,110,111,112]. In general, lipids are amphipathic or hydrophobic molecules that have been used as carriers for the delivery of active ingredients during the last decades [107]. Since the early 19th century, lipid nanoparticle drug delivery systems were considered by a German professor, R.H. Müller, and professor M. Gascon from Italy [108]. Lipid nanoparticles can be made of only solid lipids or both solid and liquid lipids [108]. The main lipids used in these nanoparticles are free fatty acids, fatty alcohols, triglycerides, steroids, and waxes [107,108]. Phospholipids, glycolipids, and sphingolipids are included as well [107]. Lipid particles are popular in drug delivery due to their biodegradability and biocompatibility, very low toxicity profile, increasing the drug solubility, and release in a controlled profile [108]. Lipid nanoparticles vary in different ways, and the main parameters in their characterization are the size, zeta potential, crystallinity rate, polymorphism, drug loading, and drug release [108]. In the case of NRG encapsulation for delivery, solid lipid nanoparticles (SLNs), nanostructured lipid carriers (NLCs), and liposomal formulations are being used [108,113]. 

Müller et al. introduced SLNs for the first time in 1991 [80]. SLNs are the first lipid nanoparticles made of solid lipids that are stabilized by emulsifiers [108]. SLNs have submicron sizes (<1000 nm). Compared to polymers, liposomes, and emulsions, they have some advantages such as low toxicity, no need to use organic solvents, controlled release of drugs, decreased chemical degradation, and low cost. Nevertheless, their crystalline structure decreases drug solubility and induces instability [80,108]. 

In one study in 2016, the researchers developed a delivery system for NRG using SLNs, to have an extended and consistent drug release, better stability, and enhanced pulmonary bioavailability [80]. NRG was incorporated into SLNs by employing emulsification and low-temperature solidification techniques. The NRG-SLNs structures were uniform spherically shaped particles with high drug loading. The results from the MTT cell viability assay on human lung epithelial cancer cell line (A549) showed that these SLNs had no toxicity. The cellular uptake of fluorescein isothiocyanate (FITC)-labeled SLNs, in A549 cells was proved to be time-dependent within 3 h. According to a pharmacokinetic study in SD rats, a considerable enhancement in bioavailability with NRG-SLNs after pulmonary administration. In conclusion, SLNs are beneficial carriers for pulmonary drug delivery systems, and these nanoparticles enhance the bioavailability of drugs such as NRG [80].

**Figure 4:**
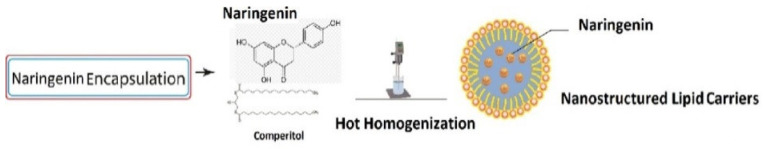
Naringenin–loaded nanostructured lipid carriers. Reproduced with permission from [114], Elsevier, 2019.

Another study investigated the effect of NRG–loaded NLCs on apoptosis induction in colon cancer cells (HT-29), along with the administration of oxaliplatin (Figure 4) [114]. NLCs are the second generation of lipid-based nanoparticles found about seven years after the SLNs [107]. NLCs are made of solid and liquid lipids able to incorporate higher drug amounts than SLN. They can enhance the solubility of drugs in the lipid matrix, and the drug release they exert is more controlled [108]. Their burst release is also lower because of their compact structure [65]. In this research, encapsulation of NRG into NLCs was done by the hot homogenization method. The nanoparticle sizes ranged between 50–120 nm (mean size = 98). To check nanoparticles cytotoxicity, the authors used an MTT assay and 4′,6-diamidino-2-phenylindole (DAPI) staining. The results showed that the toxicity of NRG-NLCs in blocking the cell growth after 24 h incubation is very similar to NRG alone. Apoptosis was induced in HT-29 colon cells by NRG and was investigated by flow cytometry assay, which represented an increase in the number of cells that went through apoptosis by using NRG-NLCs. Furthermore, based on real-time PCR results, the anti-apoptotic markers were decreased, whereas the regulation of Bid mRNA in the pro-apoptotic family was increased. According to the results of this research, NRG loaded into NLCs could successfully decrease the cell growth of HT-29 colon cancer. NRG-NLCs increase anticancer drugs’ effectiveness, reduce the side effects of chemotherapy treatments and along with chemotherapy drugs, and stimulate apoptosis [114].

Considerable attention has been paid to liposomes for decades because of their ability to target and release drugs to the target site in a controlled manner, having a desirable safety profile, being highly soluble and completely tolerated particles, which improve drug bioavailability [113,115]. Liposomes are lipid vesicles formed by phospholipids bilayers [113,115]. The lipids are often natural phospholipids such as soybean phosphatidylcholine (SPC) or synthetic phospholipids such as dipalmitoyl-phosphatidylcholine (DPPC) [116]. In 2016, Wang Y et al. evaluated the effects of loading NRG into a liposomal system for oral administration. NRG-loaded liposomes were prepared by thin-film hydration. The result showed physical stability and high zeta potential values. In vitro release profiles were obtained in three relevant gastro-intestinal media. Tissue distribution analysis showed higher drug concentrations of NRG in different tissues especially in liver, when liposomes were used. In addition, a remarkable increase in drug solubility and oral bioavailability of the encapsulated drug in rats after oral administration was seen. According to this research results, NRG-loaded liposomes can enhance drug bioavailability and solubility, being therefore suitable delivery systems for clinical applications [113].

### 4.3. Nanosuspensions

Nanosuspensions are drug delivery system that contain a pure therapeutic agent and stabilizer, causing the emergence of particles with a size <1µm. It can be an appropriate choice to solve low bioavailability and poor pharmacokinetics of insoluble drugs. It can be applied for targeted therapy and solubilization in the intravascular route and prevent enzymatic ruin in oral administration [117]. Besides the improvement of drug pharmacokinetics, its formulation is easy. NRG, such as other flavonoids such as quercetin [118], rutin [119], etc., can be prepared as nanosuspension to improve its therapeutic effectiveness. The most widely used stabilizers, surfactants, and polymers, in nanosuspensions include Tween^®^ 80, polyvinyl alcohols (PVA), pluronics (F68 and F127), polyethylene glycols (PEG), poloxamer-188, d-α-Tocopherol polyethylene glycol 1000 succinate or Vitamin E-TPGS, etc. [120]. In 2018, Kumar Singh et al. fabricated surfactant-stabilized NRG nanosuspensions to enhance its biochemical and pharmacokinetic properties [121]. They prepared different nanosuspension by precipitation-ultrasonication technique with various concentrations of diverse surfactants and polymers. They also tested several stabilizers, such as sodium cholate (SC), sodium lauryl sulfate (SLS), polyethylene glycol 4000 (PEG), Tween^®^ 80, and poloxamer-188, vitamin E-TPGS, finding the last combination, the most suitable one for NRG. TPNS improved NRG solubility because of its higher emulsification efficiency in comparison with other surfactants, monitored by micellar property of TPGS with alteration of crystalline to the amorphous form of NRG. The study revealed a considerably enriched bioavailability compared to pure NRG due to the remarkable decrease in size and the increase of solubility and dissolution. It also showed high stability for six months. 

In a similar study, TPGS nanosuspension enhanced the uptake of NRG by breast cancer cell lines and animal models, with overexpression of p glycoprotein (P-gp) [122]. P-gp is one of the significant mechanisms in multi-drug resistance (MDR) incidence in cancer cells. This transporter promotes the efflux of the drug and decreases the intracellular concentration of the drug. Some non-ionic surfactants such as TPGS can inhibit P-gp. These systems are therefore a suitable option in developing anticancer drugs to reduce MDR [123].

Additionally, the high benefit of NRG TPGS nanosuspension compared to free NRG in reducing hepatotoxicity and nephrotoxicity induced by oxidative stress after cisplatin administration was proven [124]. Rajamani et al. revealed that NRG coated with TPGS by high-pressure homogenization led to a substantial increase in its therapeutic effects and reduced liver injury by the oral route [124]. Another NRG nanosuspension was developed by Gera et al. using PVA as a stabilizer [67]. This formulation exhibited proficient NRG permeability in the gastrointestinal tract. 

In addition to surfactants, polymers can be utilized as a stabilizer for nanosuspensions. In a recent study, ten different NRG-nanosuspensions were prepared with various stabilizers and TPGS as a co-stabilizer, using a high-pressure homogenization method [125]. An improvement in oral bioavailability, dissolution, efficacy, and reduction of erythrocyte hemolysis was seen for NRG- nanosuspension. 

### 4.4. Nanoemulsions

Nanoemulsions have a high potential to modify the bioavailability of low soluble drugs through the oral route. Nanoemulsions are prepared by combining oils, surfactants, hydrophilic solvents, and co-solvents that have the unique ability to form fine oil-in-water (o/w) colloidal dispersions. To enhance the oral bioavailability and solubility of NRG, several papers reported the use of nanoemulsions as drug delivery systems for NRG [68,126,127,128]. A NRG self-emulsifying nanoemulsion was prepared with triacetin (oily phase), tween 80 (surfactant), and transcutol HP (co-surfactant) that improved NRG pharmacokinetics. High dissolution rate, optimum poly-dispersity, and rapid and complete release of the drug were achieved by optimization of this method [126].

### 4.5. Co-Delivery Systems

To consider the complexity of different diseases, one therapeutic agent is usually not sufficient; thus, a combinational therapy based on different mechanisms can improve some treatments. In 2010, Kanaz et al. prepared NRG-hesperetin (another glycan flavonoid) nanodispersion with PVP by solvent evaporation method [129]. Recrystallization of a drug in a nanocarrier can disturb the physical stability of the drug delivery system. Therefore, polymer and drug interactions can increase nanoformulation stability, especially during storage. PVP inhibits the drug crystallization by hydrogen bond formation with the drugs. Therefore, using PVP based carriers can increase formulation stability during storage. This study indicated that after three months of storage at 40 °C, the PVP nanodispersion system displayed high chemical and physical stability without any change in release profiles nor drug crystallization. Kerdudo et al. encapsulated NRG and rutin in onion-type lipid-based multilamellar vesicles (MLVs) to improve the antioxidants effects of some flavonoids, including NRG [130]. Their study demonstrated that was NRG mostly adsorbed onto the surface of nanocarriers. It showed high stability without leakage after 30 days. 

In another study, tamoxifen-NRG nanoemulsion was prepared to escalate the antitumor effects of tamoxifen against breast cancer [131]. In this nanoemulsion, polyunsaturated fatty acids (PUFAs) were used as a lipid phase. According to previous studies, PUFAs improve drug bioavailability and biodistribution. In this study, the authors fabricated nanoemulsions by a simple admixture of the formulation components. This nanoemulsion increased drug absorption and was highly effective in the suppression of tumor growth in both in vitro and in vivo studies.

In a recent study, pravastatin, an antihyperlipidemic agent for treating the atherosclerotic vascular disorder, was co-loaded with NRG in omega-3-phospholipid based nanotransfersomes [72]. This nanocarrier was developed as a transdermal drug delivery system to overcome the problems associated with the oral route administration while reducing pravastatin first-pass effects and enriching the anti-oxidant cascade through NRG. Nanotransfersomes were prepared by a modified thin-film hydration method. Omega-3-phospholipids was incorporated to improve cardiovascular and liver activities and to reduce the production of low-density lipoprotein (LDL) and triglycerides [132,133,134]. The results showed that NRG and omega-3-phospholipids, in a synergistic manner, reduced the hepatic marker enzymes levels and lipid peroxidative markers, prompted by pravastatin.

## 5. Potential Therapeutic Applications of NRG Nanoformulations

**Figure 5:**
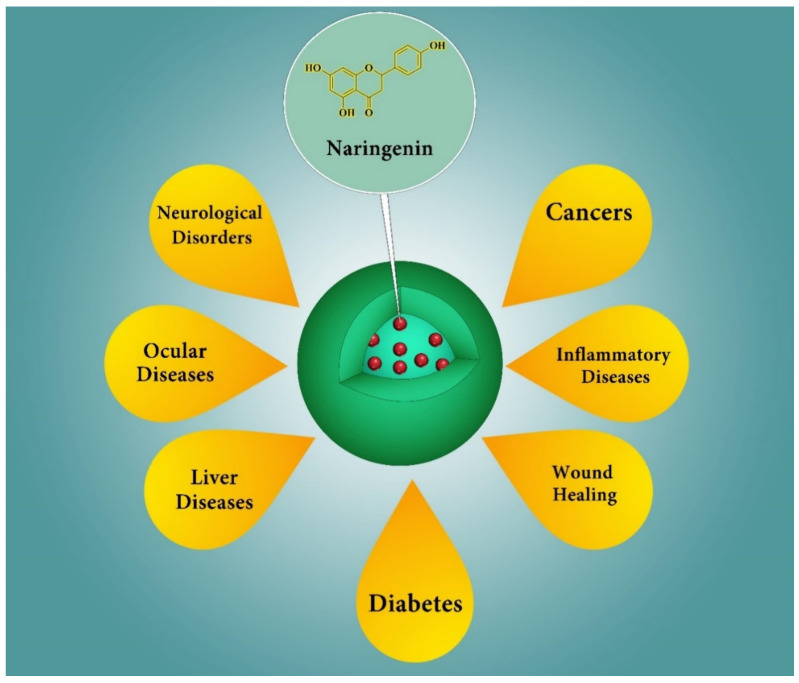
Therapeutic applications of naringenin-loaded nanocarriers.

Several potential therapeutic applications of NRG nanoformulations have been investigated in preclinical studies (both in vitro and in vivo), such as cancer prevention and treatment, brain diseases, inflammatory diseases, skin disorders, ocular diseases, liver diseases, and diabetes (Figure 5). Table 1 includes some of the most relevant applications of naringenin-loaded nanoparticles, including the particle-type. The key results are presented in comparison to free naringenin.

### 5.1. Cancer

NRG has potential cancer chemopreventive and therapeutic effects, as shown in different cell lines, such as human non-small-cell lung carcinoma (NSCLC) (A549), colorectal cancer cells (Colon-26), human cervical cancer cells (HeLa), pancreatic cancer cells, and human skin carcinoma cells (A431) [77,135,136,137,142]. NRG can induce apoptosis, reduce cancer cell viability, and induce cancer cell cycle arrest [28,143,144,145]. NRG causes carcinogen inactivation by different mechanisms. Such as, the upregulation of the enzymes uridine 5′-diphospho glucuronosyltransferase, quinone reductase, and glutathione S-transferase and the inhibition of CYP19, which helps to control breast and prostate cancer. It also presents anti-proliferative properties by downregulation of reactive oxygen species and ornithine decarboxylase, and several signal transduction enzymes. NRG inhibits cyclin, and cyclin-dependent kinase helping to control leukemia. NRG has also an angio-inhibitory effect as it decreases the vascular endothelial growth factor, that helps reduce metastasis [146]. A variety NRG nano-based drug delivery systems of different materials have been used in those studies, such as poly(d, l-lactide-*co*-glycolide) (PLGA) nanoparticles, multi-walled carbon nanotubes, silk fibroin nanoparticles, nanohybrid hydrogel, eudragit E-100 nanoparticles, polycaprolactone (PCL) nanoparticles, soluthin–maltodextrin nanocarriers, chitosan nanoparticles, and Eudragit E/PVA nanoparticles [77,135,136,137,142,147,148,149].

The cytotoxicity of NRG-loaded nanoparticles on lung cancer cells has been reported by several works. For instance, NRG chitosan particles were applied to A549 lung cancer cells. Particle cytotoxicity was evaluated by an MTT assay. The treatment with 0.5 mg/mL of NRG nanoparticles showed a significant reduction in the cancer cell viability while maintaining cell viability in normal fibroblast cells (3T3) [148]. In another approach, NRG PCL nanoparticles were decorated with hyaluronic acid as a targeting moiety. Cellular studies revealed an improved anticancer effect and uptake of NRG in A549 lung cancer cells. In animal tests, the nanoparticles showed suppressive effects on cancer growth in urethane-induced lung cancer rats [135]. Morias et al. functionalized multi-walled NRG carbon nanotubes that showed enhanced cytotoxic activity on adenocarcinomatous human alveolar basal epithelium (A569), while maintaining a safe profile in a human skin cell line (hFB) [84].

**Figure 6:**
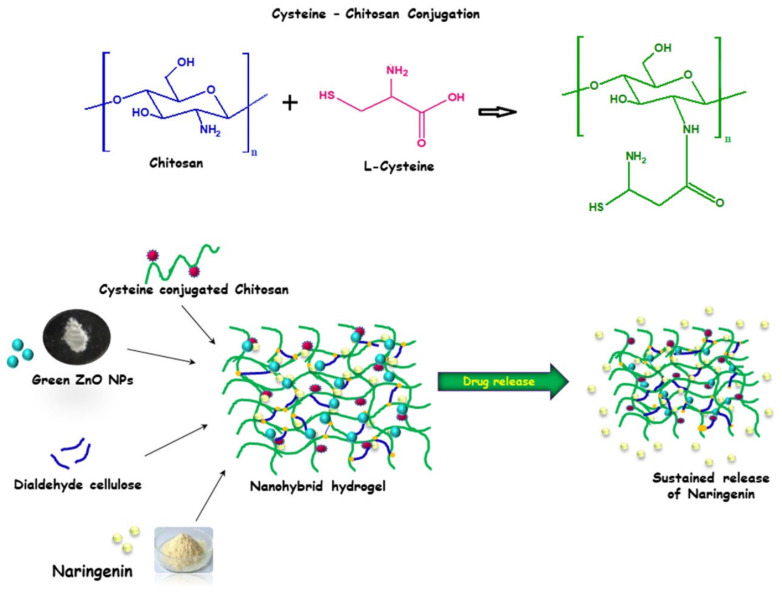
Naringenin embedded within a nanohybrid hydrogel. Reproduced with permission from [142], Elsevier, 2020.

Another novel approach was used to improve NRG delivery by employing a green nanohybrid hydrogel as a delivery system. This system was prepared by conjugation of L-cysteine (CYS) with chitosan, then phyto-synthesized zinc oxide nanoparticles were embedded to improve the electrostatic interactions between the carrier and the encapsulated drug. CYS conjugation improved the mucoadhesiveness and solubility of the system and had inhibitory effects on enzymes. Moreover, dialdehyde cellulose (DAC) was used as a green material for cross-linking the polymeric matrix to form a green nanohybrid hydrogel (Figure 6). The developed carrier achieved loading of 86.09 % of NRG with a maximum release profile of 72.78 % at a concentration of 1 mg/mL. Additionally, this system showed desirable biocompatibility when tested on L929 non-cancerous murine fibroblast cells. In contrast, the nanohybrid hydrogel showed increased cytotoxicity (two-fold) when used to treat A431 human skin carcinoma cells compared to free NRG [142].

A series of papers published by the same research group comprehensively investigated NRG-loaded nanoparticles on different occasions. In 2011, they reported the use of nanoprecipitation to prepare the NRG-loaded nanoparticles, which exhibited sustained release behavior. Furthermore, compared to free NRG, the developed nanoparticles enhanced the cytotoxicity of NRG, reduced GSH levels, increased lipid peroxidation status (TBARS), and increased reactive oxygen species (ROS) levels in human cervical cancer cells (HeLa) [79]. In their next publications, to evaluate the anti-cancer effects of these nanoparticles (dose of 50 mg/kg/day), the 7,12-dimethylbenz(a)anthracene (DMBA)-induced oral carcinogenesis model was used in Syrian hamsters. The results revealed significant suppression of histological lesions, restoration of molecular markers and biochemical parameters, decreased nucleic acid contents, anti-lipid peroxidative activity, reduced tryptophan levels, increased glycogen, inhibition of proliferation, and antioxidant activity compared to the free NRG form [149,150,151,152].

### 5.2. Brain Diseases

Neurological disorders, including brain diseases such as Alzheimer’s disease (AD) and Parkinson’s disease (PD), represent one of the most important non-communicable diseases affecting the worldwide population and are associated with significant disease burden and disability [153]. Oxidative stress is occurs naturally during the aging process, and might play an important role increasing the severity of neurodegenerative diseases such as Parkinson and Alzheimer, for which NRG has shown to be effective in reducing oxidative stress with a high permeation to the brain [146]. While the blood–brain barrier (BBB) constitutes an essential protective barrier for the central nervous system (CNS), it is often a biological barrier to successful drug delivery to the CNS because drug penetration across the BBB is dramatically restricted, rendering ineffective therapeutics. In this regard, several nanocarriers have been developed to overcome the BBB and achieve improved drug delivery, owing to their small size, diverse physicochemical composition, tailored area, and tunable features [154]. 

Ahmad et al., developed NRG-loaded nanoemulsion to improve NRG bioavailability in the brain for cerebral ischemia treatment [155]. The NRG-loaded nanoemulsion was further formulated as an in situ poloxamer-407/chitosan gel formulation, bearing thermoresponsive and mucoadhesive properties toward efficient nasal administration and displaying a gelation temperature of 28.3 °C. The obtained globules featured a size ca. 98.31 nm and a polydispersity (PDI) around 0.386. Ex vivo permeation studies conducted on bovine nasal mucosa showed slightly superiority of NRG nanoemulsion gel nanoformulation, compare to the NRG nanoemulsion nanoformulation, and a substantial increase in NRG permeation compared to NRG suspension after a period of 8 h. Intranasal administration of the NRG nanoemulsion gel also substantially increased NRG brain concentration, the locomotor activity of rats, and its anti-oxidant and neuroprotective activities. Intranasal drug delivery represents a non-invasive therapeutic approach that allows proximity and accessibility to the central nervous system while reducing systemic exposure and bypassing the BBB. A significant reduction in infarct volume observed in stroke disease rat models with NRG suspension was also observed. The NRG nanoemulsion gel showed to be a safe, effective, and non-invasive strategy for NRG brain delivery for the treatment of cerebral ischemia [155].

The neuroprotective properties of NRG were also explored as a therapeutic strategy to tackle other brain diseases. Shadab et al. optimized NRG-loaded nanoemulsions with a droplet size ca. 114 nm, a zeta potential around 12.4, and a PDI of ca. 0.312 to ascertain NRG-mediated neuroprotective activities to fight AD [156]. NRG nanoemulsion was prepared from capryol 90 as oily phase, tween 20 as a surfactant, and ethanol as a co-surfactant. The in vitro neuroprotective properties of the NRG- loaded nanoemulsion showed enhanced NRG neuroprotective properties in Aβ-induced SH-SY5Y neuronal cells and decreased Aβ levels compared with free NRG. A reduced NRG dose was required to exert the same reduction in ROS activity and down-regulation of amyloidogenesis-related genes, as evidenced by decreases in protein levels of total and phosphorylated tau (pT231), amyloid precursor protein, and β-secretase. Further studies are needed to demonstrate the in vivo neuroprotective applications of NRG nanoemulsions as a potential AD treatment [156]. 

In another study, Shadab et al. applied the neuroprotective and anti-oxidant properties of NRG to PD treatment [81]. Oxidative stress has been implied in PD pathogenesis and has emerged as one of its leading causes, accounting for significant neurotoxicity and neuronal death for which NRG anti-oxidant and neuro-protective properties are of great interest. NRG was loaded in positively-charged chitosan nanoparticles (size ca. 88 nm, PDI ca. 0.31, zeta potential ca. 15.36) prepared by the ionic gelation method, eliciting high NRG encapsulation efficiency (>91%) and intended for nose-to-brain delivery. Ex vivo nasal permeation studies showed a two-fold improvement on NRG permeation by the NRG-loaded nanoparticles compared to the free-NRG solution after 20 h, possibly helped by the electrostatic interactions between amine groups of chitosan and negatively-charged sialic acid groups present on cell membranes, thus facilitating paracellular NRG transport and permeation through the nasal epithelium. Results obtained for the NRG-loaded NPs in 6- SH-SY5Y cells as cell model evidenced hydroxydopamine (6-OHDA)-induced neurotoxicity. Results showed improved in vitro antioxidant properties and an improved ability to reduce ROS levels. The highest neuroprotective effect was measured through cell viability measurements, revealing interesting properties toward nano-based nose-to-brain NRG delivery for PD management [81]. 

Another work investigated NRG-loaded Vitamin E-based NE, composed of capryloyl 90: vitamin E as oily phase and tween 80 as a surfactant, for NRG intranasal delivery. Possible synergistic effects of the anti-oxidant activities of vitamin E and NRG were evaluated [138]. The optimized nanoemulsion displayed a droplet size of ca. 38 nm, a PDI <0.15 and negative zeta potential (ca. −27). In vitro studies comparing NRG nanoemulsion and NRG suspension showed significantly higher drug release by NRG nanoemulsion. Ex vivo studies in goat nasal mucosa showed a three-fold increase in permeation for the NRG nanoemulsion. In vivo models elicited increased biodistribution and bioavailability of NRG in the brain after intranasal administration of NRG nanoemulsion, compared with administration of intravenous NRG nanoemulsion and intranasal NRG solution. Considerable anti-oxidant effects and successful improvement in the behavioral conditions were obtained in 6-OHDA-pretreated rats, with the highest performance in combination with standard oral levodopa therapy [138].

### 5.3. Inflammatory Diseases

In the case of occurrence infection or injury, macrophages produce cytokines (e.g., IL-1β, IL-6, TNF-α) and other mediators such as nitric oxide and prostaglandins during inflammatory processes. Excess production of these compounds leads to many characteristic chronic inflammatory diseases such as asthma, atherosclerosis, osteoarthritis, pulmonary fibrosis, rheumatoid arthritis, septic shock, tumor etc. Therefore, their inhibition is a crucial target pathway in the management of many chronic inflammatory diseases [157,158,159]. Hydroxyl substituents (OH) on NRG chemical structures are responsible for its antioxidant acitivity by having a high reactivity agains ROS and reactive nitrogen species (RNS). NRG has an excellent ability to scavenge free radicals and to prevent lipid peroxidation-mediated oxidative DNA damage, which can be exploited to treat inflammatory diseases [160]. In this context, Kumar and Abraham designed a PVP-coated NRG nanoparticle. Biocompatibility and immunomodulatory effects, as well as safety of these nanoparticles for diagnostic and therapeutic applications compared with AgNPs were confirmed through in vivo toxicity studies in male SD rats [44]. The objective of their next study was to investigate the inhibitory effects of NRG on lipopolysaccharide-induced inflammation in RAW264.7 mouse macrophage cells [139]. In brief, the study results indicated that the NRG nanoparticle had a high potential to reduce the levels of inflammatory mediators. It suppressed the expressions of mediator-producing enzymes and the pro-inflammatory cytokines. 

**Figure 7:**
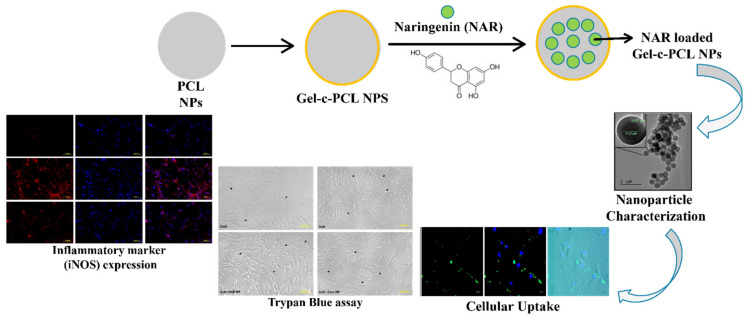
NRG-loaded gelatin-coated polycaprolactone to ameliorate oxygen glucose deprivation-induced inflammatory stress on human mesenchymal stem cells. Reproduced with permission from [161], ACS publications, 2018. Abbreviations: iNOS, inducible nitric oxide synthase; NAR, naringenin; NPs, nanoparticles; PCL, polycaprolactone.

Ischemic stroke may lead to a systemic inflammatory response with the access of blood components to the brain parenchyma through the possible breakdown of the blood–brain barrier. In this regard, anti-inflammatory compounds such as NRG are viable candidates to protect against ischemic stroke and other related disorders. In this regard, Ahmad et al. investigated the effect of NRG entrapped in gelatin-coated PCL nanoparticles on human mesenchymal stem cells (Figure 7) [161]. The observed reduction in pro-inflammatory cytokine and other inflammatory biomarker levels indicated that NRG nanoparticles exert neuroprotective effects on these cells by suppressing the oxygen-glucose deprivation-induced inflammatory responses. Thus, their nanoformulation could offer a promising therapeutic potential against ischemic stroke and other neuro-inflammatory diseases.

Rajamani et al., suggested a nanosuspension as a preferred dosage form to increase the solubility of NRG and protect it from degradation. They prepared the formulation by the high-pressure homogenization method [125]. In a subsequent study, in vitro and in vivo studies were performed to evaluate the anti-inflammatory activity of the nanosuspension compared to standard drug and free NRG [162]. Significantly higher therapeutic and anti-inflammatory activity of the NRG, even using a lower concentration, was observed compared to normal NRG and diclofenac sodium.

### 5.4. Topical Applications

Several authors have also studied the activity of NRG in treating various skin diseases and protecting the skin from ultraviolet (UV) irradiation [78]. The excess of ROS in the skin contributes to the development of several cutaneous diseases [78]. The anti-inflammatory and immunomodulatory activities of NRG predict its potential efficacy in dermatological diseases associated with immunological components, such as psoriasis or atopic dermatitis. For instance, NRG suppress NF-κB therefore it inhibits IL-33, TNF-α and IL-1β in paw skin inflammatory pain [19].

Tae-Ho Kim et al., demonstrated its efficacy in the treatment of atopic dermatitis induced by 1-fluoro-2,4-dinitrobenzene (DNFB) in NC/Nga mice. In this study, NRG was administered intraperitoneally at a dose of 50 or 100 mg/kg daily for one week. The authors reported reduced ear swelling and improved dorsal skin lesions compared to the control [163].

Gaggeri and colleagues demonstrated that the S-enantiomer of NRG has TNF-α blocking activity [164,165]. Based on this work, Chlapanidas et al. developed a sericin microparticle system containing NRG and tested its efficacy in vitro in the LPS-stimulated human peripheral blood mononuclear cell (hPBMC) model [166]. The authors demonstrated higher efficacy of the NRG particles than the free drug control, which can be attributed to the additional sericin TNF-α blocker effect and the improved bioavailability by the particles.

NRG is also effective against oxidative skin damage. Ming-Jun Tsai and colleagues developed an NRG-loaded submicron emulsion system for topical application and studied the permeability of the active ingredient through the skin, stability, and skin irritation in vivo. The particles were stable, maintaining 98% of the active ingredient after 3 months of storage at 25 °C and 40 °C. The transdermal and biodistributed amounts of NRG in the skin of abdominal SD rats were significantly increased about (4.5–9.4 times) compared with a saturated aqueous NRG solution. The irritation test showed that the NRG-loaded submicron triggered lower irritation signs than the negative control group (treated with water) [167]. 

The same authors later developed flexible liposomes to enhance NRG permeability through the skin [78]. Flexible liposomes are lipid-based vesicles that usually contain surface-active ingredients; in this case, Tween^®^ 80. They can encapsulate hydrophilic drugs in their aqueous core or lipophilic drugs in their phospholipidic bilayer. These vesicles increased NRG solubility and allowed the application of higher doses. The authors demonstrated that the chemical enhancer tween 80 and the flexible liposomes increased NRG SD rats abdominal skin permeability compared to a saturated control solution at similar levels. Interestingly, the liposomes increased the deposited amount of NRG 1.2-1.9-fold compared with the Tween^®^ 80 solution, being highest for the liposomes, including the highest amount of Tween^®^ 80. The authors relate this improvement to the elasticity of the vesicles and their ability to squeeze between the corneocytes, thus favoring the passage of NRG [78]. However, the authors do not provide elasticity data of the vesicles. This would have been of major interest to draw this conclusion, as liposomes were prepared, including cholesterol, which stabilizes the lipid membrane, making the bilayer more rigid [168]. The same can be said for NRG, whose solubility places it in the bilayer more than in the aqueous core, as observed in vitamin D3-liposomes [169]. Dreier et al.s’ work demonstrated by super-resolution and fluorescence dynamics that liposomes do not cross the human skin barrier remaining intact [170]. Tsai and coworkers also provided an in vivo skin irritation experiment, concluding that the liposomal formulation did not trigger local irritation and is safe for topical delivery [78].

Through in vivo studies, Martinez et al., demonstrated the activity of NRG to prevent UV-B irradiation-induced inflammation and oxidative stress in the skin; therefore, they developed a topical formulation containing NRG [171,172]. Based on these works, Badea et al. developed NLCs containing NRG. The UV-A filter diethylamino hydroxybenzoyl hexyl benzoate (DHHB) and the combination of NRG and DHHB in solid lipid + vegetable oils-core nanoparticles [173]. Their objective was to retain the UV filter onto the skin for a more extended period to physically block the UV rays while enhancing the permeability of the antioxidant to the deeper skin layers. Here, the flavonoid can neutralize the ROS formed, thus avoiding cellular and DNA damage. Authors compared the particles with freeze-dried emulsions containing the same amounts of the particle excipients but were not subjected to homogenization procedures. Particles and emulsions were further incorporated in carbopol 940 hydrogels to allow topical application. The authors showed that the presence of NRG increased the absorption properties of the nanoparticles in the UVA range 320-360 nm, also when incorporated into the hydrogels. NRG-loaded capsules also were the most effective systems against ROS and 2,2′-Azino-bis(3-ethylbenzthiazoline-6-sulfonic acid (ABTS) radical cation. The authors also showed in vitro cytotoxicity on L929 murine fibroblasts using an MTS-test. Cell viability was significantly higher for NRG-loaded capsules compared to the other capsules. Release studies showed controlled release of NRG and DHHB, allowing a slow release of the UV filter and a much faster release of NRG, as desired. The permeability study was performed using a skin surrogate: a collagen membrane, showing a higher NRG permeability when formulated into the capsules compared to the emulsion [173].

Haritima Joshi and coworkers also studied the sunscreen performance of NRG-loaded PLGA nanoparticles, using in vitro and in vivo methods [174]. The authors demonstrated the absence of cytotoxicity of the NRG free drug and the loaded nanoparticles using HaCaT cells and an MTT test, and the absence of skin toxicity using an in vivo model in Wistar rats. Skin permeability was assessed using Franz Diffusion Cells with rat skin, and drug retention studies were also performed. Permeability tests showed an increased permeability compared to free drug, and the accumulation of NRG within the diffusional membrane was also significantly higher. They attribute the increased permeability to the ability of nanoparticles to penetrate the skin, but this is not supported by data, and there is no bibliographic evidence of intact PLGA-nanoparticle penetration through the skin (particle size >5 nm), up to our knowledge. When incorporating NRG free drug and nanoparticles, the authors showed the contrary effects. These data support the general agreement that nanoparticles release the drug in a controlled manner, reducing skin permeability, even though the authors do not show release data. Additionally, the authors forgot the standard deviations in the permeability profiles. Some of them do not seem to have reached the steady state from which the permeability parameters can be calculated. This work is promising and supports the feasibility of administering NRG via the skin using PLGA-nanoparticles. However, there is no evidence from this work to improve NRG permeability or performance [174]. Further investigations should be conducted to elucidate if these administration systems provide advantages over other drug delivery systems or plain drugs.

From the reviewed works, it appears that NRG deserves deeper exploration of its potential cutaneous applications, as it remains to be proven whether such activity can be harnessed for skin diseases. Moreover, no research has been conducted on human skin, which can be an important source of variability in NRG permeability behavior. Nevertheless, the use of nanotechnology in topical products seems promising to improve the delivery of NRG across the skin for different protective and therapeutic applications.

### 5.5. Ocular Applications

Recently, interesting pharmacological activities have been attributed to NRG, such as anti-oxidant activities, which have applications in the treatment of various ocular diseases. Nano-based strategies to improve topical ocular drug delivery include increasing the solubility of poorly soluble drug, prolonged and controlled release of drug, increasing ocular bioavailability and stability of drugs, dose reduction, and facile administration [2,6,15,18,21,22]. NRG has shown different effects on ocular diseases such as, prevention of retinal damage in diabetic retinopathy, inhibition of corneal neovascularization, and anti-inflammatory effect on uveitis [175,176,177].

Wang et al., developed PVP K-17PF/NRG nanocomplexes aimed at ocular delivery of NRG to investigate the ocular anti-inflammatory activities of NRG [82]. PVP, a widely used and highly biocompatible water-soluble polymer with pronounced ocular tolerance, acted as a hydrophilic NRG complexing agent. It is able to form intermolecular cross-links with hydrophobic compounds such as NRG, thus increasing the water solubility of poorly soluble compounds. In addition, it can enhance the viscosity and stability of ophthalmic formulations or act as a suspending agent in suspensions and solutions for ocular delivery. The nanocomplexes were prepared via a simple thin-layer hydration technique and showed complexation efficiency of about 98.51, a mean diameter of approximately 6.73 nm, and PDI around 0.254. In vitro studies in human corneal epithelial cells (HCECs) showed that the ophthalmic dispersion of the nanocomplexes displayed good tolerability, both short- and long-term cytocompatibility, improved anti-oxidant activity, and over 10-fold enhanced NRG membrane permeation compared to free NRG in a membrane permeability assay. The ophthalmic dispersion containing K-17PF/NRG nanocomplexeswas further tested topically in New Zealand white rabbits. The results showed good tolerability, improved intraocular permeation of NRG, and improved biodistribution in ocular tissue, namely almost five times higher concentrations in retina tissue compared to free NRG, as well as improved anti-inflammatory activity in sodium arachidonate-induced inflamed rabbit eyes [82]. Another study by Wang et al. reported NRG-loaded sulfobutylether-β-cyclodextrin/chitosan nanoparticles for ocular NRG delivery and treatment of age-related macular degeneration (AMD), a progressive ocular tissue disease responsible for irreversible impairment and loss of vision [83]. NRG was loaded into sulfobutylether-β-cyclodextrins forming stable guest-host complexes capable of improving NRG’s solubility. Chitosan, one of the most extensively used biodegradable and biocompatible biopolymers, can interact with negatively-charged sulfobutylether-β-cyclodextrin by an ion gelation method, assembling sulfobutylether-β-cyclodextrin/chitosan nanoparticles. The results showed a particle size of ca. 450 nm and a positively charged surface (zeta potential ca. +22.5), which favored interaction with negatively charged cornea tissue. The particles enhanced the ocular residence time as well as the concentration and corneal penetration of NRG. Topical ophthalmic delivery of NRG in sulfobutylether-β-cyclodextrin/chitosan nanoparticles demonstrated sustained NRG release in simulated tear fluid and good ocular tolerance in rabbit eyes in an in vivo assay with New Zealand white (Draize test). Their work demonstrated an improvement in ophthalmic bioavailability of NRG, evidencing suitable features for improved ocular delivery of hydrophobic drugs and treatment of eye disorders [83].

### 5.6. Liver Diseases

NRG, as a well-known natural compound with anti-oxidant, anti-inflammatory, and anti-tumor properties has been comprehensively researched for its potential beneficial effects in liver diseases with low toxicity. Liver diseases are caused by different etiological agents, mainly drug intoxication, alcohol consumption, malnutrition, or viruses [14]. Liver disorders are often induced by a combination of oxidative stress and inflammation. Therefore, the demonstrated activities of NRG may be of great interest in the prevention and treatment of liver diseases. NRG can inhibit oxidative stress, toll-like receptor (TLR), the mitogen-activated protein kinase (MAPK), and non-canonical TGF-β pathways. NRG has been shown to have activity in non-alcoholic fatty liver disease, as it can regulate lipid metabolism by modulation the synthesis and oxidation of lipids and cholesterol [14]. 

NRG has been studied in various in vivo and in vitro liver injury models, using hepatic injury agents such as *N*-methyl-*N*-nitro-nitroguanidine, lipopolysaccharide (LPS), or carbon tetrachloride (CCl4), among others [14]. 

NRG activity against hepatic and renal injury caused by antituberculosis was investigated in rats. The authors concluded from the work that the pharmacological action of NRG at 20 and 40 mg/kg dose was equally effective as positive control silymarin without adverse effects in oxidative stress and antioxidant activity [178]. The therapeutic efficacy of silymarin and naringenin in reducing arsenic-induced hepatic damage in young rats was also studied. Arsenic can cause hepatic injury directly binding with –SH groups or indirectly through generation of reactive oxygen species (ROS). The phenolic structures, have antioxidant effect and inhibit free radical-mediated processes. The author’s results showed that chronic arsenic exposure significantly increased blood ROS and a reduction in blood GSH levels, supporting the hypothesis of arsenic-induced oxidative stress. Both silymarin and naringenin administration reversed this trend, demonstrating antioxidant properties of both the flavonoids through s SOD, catalase and GPx activities protection, by directly scavenging ROS while inhibiting lipid peroxidation [179].

Different nanotechnological approaches to improve drug bioavailability have also been tested to improve NRG access to the liver. In the case of NRG, Yen et al. investigated its protective effect in the CCl4-induced acute liver failure model by administering NRG orally as a free drug and in Eudragit^®^ E 100 nanoparticles [180]. The particles were prepared by nanoprecipitation technique and stabilized with PVA. The authors pre-treated the animals with the formulations prior to CC14 administration. The authors examined plasma levels of aspartate aminotransferase (AST) and alanine aminotransferase (ALT) and measured the anti-oxidant enzymes and lipid peroxidation and the caspase-3, -8, and -9 activities in liver tissue homogenates. The authors demonstrated a particle-mediated increase in NRG solubility and controlled drug release. Pre-treatment of animals with the drug and the loaded particles reduced plasma levels of AST and ALT by 1-1.5 fold compared with the control. Regarding the anti-oxidant enzyme production, the authors found a significant increase of superoxide dismutase, catalase, and glutathione-peroxidase levels when the animals were treated compared with the control. They found 2–2.3-fold superior amounts when NRG was encapsulated compared with treatment with the free drug. Caspases 3 and 9 were significantly suppressed by free NRG, and the 3 analyzed caspases were suppressed by the encapsulated drug. These results show a clear improvement of NRG delivery via the oral route with an increased dose reaching the target site, thus improving its efficacy [180]. 

Liposomes present several interesting properties from the drug delivery point of view, as they are completely biocompatible and easily biodegradable. Wang et al., prepared NRG loaded liposomes to improve oral drug bioavailability [113]. Their pharmacokinetics in in vivo studies showed an increase in the AUC area of the NRG-loaded liposomal formulation. The authors developed from 16648.48 to 223754.0 ng/mL−1 h compared to the pure drug in mice after oral administration. Furthermore, this work showed that NRG distribution was higher in different tissues for the liposomes than the pure drug, being significantly more predominant in the liver [113]. 

Chen and coworkers investigated the efficacy of NRG-loaded liposomes on the nonalcoholic fatty liver disease (NAFLD) in mice [140]. The authors designed NRG-loaded liposomes, studied their release profile and the pharmacokinetics in vivo, compared to free drug. They also designed an animal model of NAFLD in C57BL/6J mice by removing methionine choline from the diet. This work showed effective drug encapsulation and uniform drug distribution with a sustained release profile in vitro. The pharmacokinetic experiment demonstrated improved oral bioavailability of NRG when encapsulated in liposomes, supporting the findings reported by wang and coworkers [113]. The in vivo experiment with NAFLD mice showed hepatic lipid reduction with better hepatocyte protective properties than the controls, demonstrating the improvement of NRG administration and activity thanks to encapsulation in liposomes [140].

Although the hepatoprotective effects of NRG have been widely investigated, only a few works can be found to deliver NRG precisely to the liver. As can be concluded from the works mentioned above, NRG delivery and, therefore, its hepatoprotective effects are significantly higher when administered in nanomedicines. Further investigations are needed to understand better the underlying mechanisms of this improvement and drug-particle-target interactions.

### 5.7. Diabetes

NRG has demonstrated anti-hyperglycemic activity by preventing glucose absorption from the intestine. The mechanism of NRG anti-diabetic effects are attributed to increasing insulin sensitivity, decreasing glucose blood levels, suppressing the macrophages migration into adipose tissue, inhibiting monocyte chemoattractant protein-1 (MCP-1), decreasing insulin receptor substrate-2 (IRS-2) levels, increasing phosphoinositide 3-kinase (PI3K) activity [146]. However, as explained above, its extreme water insolubility, rapid biodegradation, and low bioavailability restrict its oral administration. Nanotechnology-based drug delivery systems could be utilized to overcome these limitations. In this approach, NRG is encapsulated in polymeric nanoparticles to protect the drug from enzymatic and hydrolytic degradation in the gastrointestinal tract, which improves its availability.

**Figure 8:**
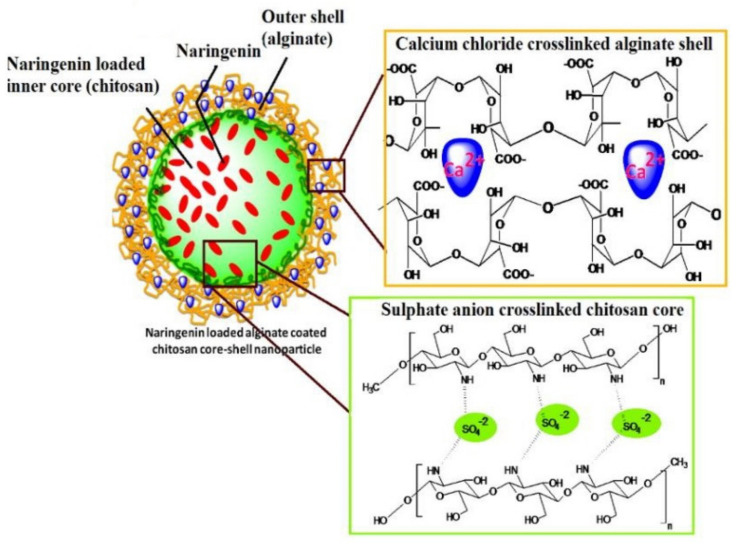
Alginate coated chitosan coreshell nanocarrier system for naringenin delivery. Reproduced with permission from [181], Elsevier, 2017.

In this context, a nanovehicle was synthesized from bio-safe and cost-effective polymers. The proposed alginate-coated chitosan core-shell nanocarrier system was safe in terms of the process conditions, including no harsh chemicals (Figure 8). Its structural chemistry also resulted in nanoparticles with reduced size and significant encapsulation efficiency (>90%). Mucoadhesion analysis and release studies exhibited pH-responsive, slow and sustained release of the NRG from the prepared nanoformulations. According to in vivo studies results, a significant hypoglycemic effect was observed in streptozotocin-induced diabetic rats after oral administration of NRG-loaded nanoparticles. Furthermore, histopathology and blood parameters revealed that the polymeric nanoparticles’ oral administrations were free from systemic toxicity [181].

In a recent study, Maity and Chakrabort prepared PLGA nanoparticles loaded with NRG by the emulsion–diffusion–evaporation technique. The NRG entrapment was approximately 70%, and the nanoparticles obtained size of about 129 nm. Furthermore, they compared the anti-diabetic potential of NRG-loaded PLGA nanoparticles compared with free NRG in streptozotocin-induced diabetic rats. According to their findings of in vivo studies, a significant decrease in glycated hemoglobin level, elevation in insulin level, and enhancements in dyslipidemia and oxidative stress parameters were observed in the nanoparticle-treated rats, but not in the rats treated with free NRG [141].

## 6. Conclusions and Future Directions

Several pre-clinical studies suggest NRG potential use against a wide range of diseases due to its broad-spectrum pharmacological effects both in vitro and in vivo. However, its low solubility in water and low oral availability limits the use of this natural product in pre-clinic and clinical research studies. Nanotechnology has proven to be an efficient way to improve the availability of NRG through different delivery routes and to enhance its efficacy in the treatment of cancer, inflammation, diabetes, liver, brain, and ocular diseases through several in vitro and in vivo methods. There are several potential therapeutic applications of pure NRG that could still be improved using NRG-loaded nanoparticles, such as: infectious, pulmonary, and cardiovascular diseases. Despite that pure NRG has already been studied in a limited number of clinical trials, there is still a need to further investigate the free drug and the NRG-loaded nanosystems in humans. Gaining knowledge about the interactions with the human body is still necessary to translate these nanoformulations to pharmaceutical products nutraceutical supplements.

## Figures and Tables

**Table 1:** Some of the potential therapeutic applications of naringenin-loaded nanoparticles and their advantages compared to pure naringenin.

Potential Therapeutic Application	Platform	Dose (Model)	Effects on Biological Parameters	Key Results	Reference
Lung cancer	Polymeric nanoparticles	50 mg/kg (urethane-induced lung cancer in rat albino wistar rat and A549 lung cancer cells and)	IC50 of 5.33 µMNRG-loaded nanoparticle compared with 25.1 µM for free NRG in A549 lung cancer cells.	NRG-loaded PCL nanoparticles decorated with hyaluronic acid were able to enhance the anticancer effect and cellular uptake of NRG in lung cancer cells nanoparticles and to suppress tumor growth in rats with urethane-induced lung cancer.	[135]
Colorectal cancer	Polymeric nanoparticles	40 mg/kg (murine colon-26 tumor-bearing BALB/c mice)	Mice survival rates were 83.33% and 33.33% for NRG-loaded nanoparticles and free NRG, respectively.	NRG delivered with eudragit E-100 nanoparticles showed a significantly higher bioavailability, enhanced cytotoxicity in cancer cells, and significantly inhibited tumor growth and enhanced survival rate in mice-bearing colorectal tumors.	[136]
Pancreatic cancer	Polymeric nanoparticles	0-60 µM (pancreatic cell line)	After 72 h, the IC50 was 32.08 µg/mL and 73.2 µg/mL for NRG-loaded nanoparticles and free NRG, respectively.	Treatment with NRG-loaded PLGA nanoparticles showed increased cytotoxicity in pancreatic cancer cells compared to NRG alone.	[137]
Parkinson’s disease	Nanoemulsion	40 mg/mL (6-OHDA induced Parkinson’s disease in rats)	The antioxidant activity (DPPH test) of NRG nanoemulsion was 95.28 ± 0.64 % compared with 78.32 ± 0.81 % for free NRG	Intranasal administration of NRG nanoemulsion and levodopa lead to reversing Parkinson’s disease symptoms in rats.	[138]
Lipopolysaccharide-induced inflammation	Polymeric nanoparticles	10 µg/mL to 200 µg/mL (LPS induced RAW264.7 cells)	Nitrite levels at a conceteration of 25 µg/mL of NRG were significantly lower when NRG-loaded nanoparticles were used compared with free NRG.	PVP-coated NRG nanoparticles showed anti-inflammatory effects on lipopolysaccharide-induced inflammation in RAW264.7 macrophage cells through the downregulation of iNOS and COX-2 expression after inhibiting MAPK and NF-κB pathways.	[139]
Wound healing	Nanoemulsion	2 mg/mL once daily for 14 days (albino Wistar rats with abrasion wound)	NRG-loaded nanoemulsion showed mild to moderate penetration of inflammatory cells infiltration into the dermis, whereas the blank formulation showed acanthosis and infiltration of inflammatory cells into the dermis	Chitosan-coated NRG nanoemulsion showed a controlled release profile and significantly ameliorated the wound’s construction and stimulated the skin regeneration.	[68]
Ocular inflammation	Nanocomplexes	5 mg/mL (New Zealand white rabbits)	conjunctiva swelling, congestion, and iris hyperemia were milder when NRG nanocomplexes were used compared with free NRG and control.	NRG PVP nanocomplex dispersion had better antioxidant properties and was well-tolerated with a significant improvement in terms of anti-inflammatory effects and intraocular permeation in the rabbit eyes.	[82]
Nonalcoholic fatty liver	Liposomes	25 mg/ kg/day for NRG-loaded liposomes and 25, 50, 100 mg/ kg/day for free NRG (nonalcoholic fatty liver in male C57BL/6J mice)	NRG-loaded showed significantly better effects on AST, ALT, TG, and lipid accumulation compared with free NRG at the same dose; however, NRG-loaded liposomes showed comparable effects to free NRG at a dose of 100 mg/ kg/day.	Nanoliposomes loaded with NRG significantly improved the oral bioavailability of NRG and showed hepatoprotective effects in mice with nonalcoholic fatty liver disease with lower effective doses compared to NRG alone.	[140]
Diabetes	Polymeric nanoparticles	25 mg/kg (streptozotocin-induced diabetic rats)	After treatment blood glucose levels were 83.3 ± 6.0 mg/dL and 126.4 ± 5.1 mg/dL for NRG-loaded nanoparticles and free NRG, respectively.	NRG-loaded PLGA nanoparticles normalized the blood glucose level in diabetic rats, increased insulin levels, reduced glycated hemoglobin level, ameliorated oxidative stress, hyperlipidemia, and hyperglycemia.	[141]

NRG: naringenin; PCL: polycaprolactone; GI50: 50% cell growth inhibition; PLGA: poly(lactic-*co*-glycolic acid); IC50: half maximal inhibitory concentration; DPPH: 2,2-diphenyl-1-picrylhydrazyl; 6-OHDA: 6-hydroxydopamine; AST: aspartate transaminase; ALT: alanine transaminase; TG: triglyceride; PVP: polyvinylpyrrolidone; iNOS: inducible nitric oxide synthase; COX-2: cyclooxygenase-2; MAPK: mitogen-activated protein kinase; NF-κB: nuclear factor kappa-light-chain-enhancer of activated B-cells.

## References

[1] Zobeiri M., Belwal T., Parvizi F., Naseri R., Farzaei M.H., Nabavi S.F., Sureda A. (2018). Naringenin and its Nano-Formulations for Fatty Liver: Cellular Modes of Action and Clinical Perspective. Curr. Pharm. Biotechnol..

[2] Patel K., Singh G.K., Patel D.K. (2014). A Review on Pharmacological and Analytical Aspects of Naringenin. Chin. J. Integr. Med..

[3] Nouri Z., Fakhri S., El-Senduny F.F., Sanadgol N., El Ghani A., Farzaei M.H., Chen J.-T. (2019). On the Neuroprotective Effects of Naringenin: Pharmacological Targets, Signaling Pathways, Molecular Mechanisms, and Clinical Perspective. Biomolecules.

[4] Cavia-Saiz M., Busto M.D., Pilar-Izquierdo M.C., Ortega N., Perez-Mateos M., Muñiz P. (2010). Antioxidant Properties, Radical Scavenging Activity and Biomolecule Protection Capacity of Flavonoid Naringenin and Its Glycoside Naringin: A Comparative Study. J. Sci. Food Agric..

[5] Tripoli E., Guardia M.L., Giammanco S., Majo D.D., Giammanco M., NBSP (2007). Citrus Flavonoids: Molecular Structure, Biological Activity and Nutritional Properties: A Review. Food Chem..

[6] Nagula R.L., Wairkar S. (2019). Recent Advances in Topical Delivery of Flavonoids: A Review. J. Control Release.

[7] Gattuso G., Barreca D., Gargiulli C., Leuzzi U., Caristi C. (2007). Flavonoid Composition of Citrus Juices. Molecules.

[8] Llorach R., Martínez-Sánchez A., Tomás-Barberán F.A., Gil M.I., Ferreres F. (2008). Characterisation of Polyphenols and Antioxidant Properties of Five Lettuce Varieties and Escarole. Food Chem..

[9] Vallverdú-Queralt A., Odriozola-Serrano I., Oms-Oliu G., Lamuela-Raventós R.M., Elez-Martínez P., Martín-Belloso O. (2012). Changes in the Polyphenol Profile of Tomato Juices Processed by Pulsed Electric Fields. J. Agric. Food Chem..

[10] Wang H., He Y., Hou Y., Geng Y., Wu X. (2020). Novel Self-Nanomicellizing Formulation Based on Rebaudioside A: A Potential Nanoplatform for Oral Delivery of Naringenin. Mater. Sci. Eng. C.

[11] Joshi R., Kulkarni Y.A., Wairkar S. (2018). Pharmacokinetic, Pharmacodynamic and Formulations Aspects of Naringenin: An Update. Life Sci..

[12] Venkateswara Rao P., Kiran S., Rohini P., Bhagyasree P. (2017). Flavonoid: A Review on Naringenin. J. Pharmacogn. Phytochem..

[13] National Center for Biotechnology Information PubChem Compound Summary for CID 439246, Naringenin. https://pubchem.ncbi.nlm.nih.gov/compound/Naringenin.

[14] Hernández-Aquino E., Muriel P. (2018). Beneficial Effects of Naringenin in Liver Diseases: Molecular Mechanisms. World J. Gastroenterol..

[15] Ahmed S., Khan H., Aschner M., Hasan M.M., Hassan S.T. (2019). Therapeutic Potential of Naringin in Neurological Disorders. Food Chem. Toxicol..

[16] Dou W., Zhang J., Sun A., Zhang E., Ding L., Mukherjee S., Wei X., Chou G., Wang Z.-T., Mani S. (2013). Protective Effect of Naringenin against Experimental Colitis via Suppression of Toll-like Receptor 4/NF-κB Signalling. Br. J. Nutr..

[17] Salehi B., Fokou P.V.T., Sharifi-Rad M., Zucca P., Pezzani R., Martins N., Sharifi-Rad J. (2019). The Therapeutic Potential of Naringenin: A Review of Clinical Trials. Pharmaceutics.

[18] Hartogh D.J.D., Tsiani E. (2019). Antidiabetic Properties of Naringenin: A Citrus Fruit Polyphenol. Biomolecules.

[19] Zeng W., Jin L., Zhang F., Zhang C., Liang W. (2018). Naringenin as a Potential Immunomodulator in Therapeutics. Pharmacol. Res..

[20] Zaidun N.H., Thent Z.C., Latiff A.A. (2018). Combating Oxidative Stress Disorders with Citrus Flavonoid: Naringenin. Life Sci..

[21] Orhan E.I., Nabavi S.F., Daglia M., Tenore G.C., Mansouri K. (2015). Naringenin and Atherosclerosis: A Review of Literature. Curr. Pharm. Biotechnol..

[22] Brewer M.S. (2011). Natural Antioxidants: Sources, Compounds, Mechanisms of Action, and Potential Applications. Compr. Rev. Food Sci. Food Saf..

[23] Jung H.A., Paudel P., Seong S.H., Min B.-S., Choi J.S. (2017). Structure-Related Protein Tyrosine Phosphatase 1b Inhibition by Naringenin Derivatives. Bioorganic Med. Chem. Lett..

[24] Pannu A., Goyal R.K., Ojha S., Nandave M. (2019). Naringenin: A Promising Flavonoid for Herbal Treatment of Rheumatoid Arthritis and Associated Inflammatory Disorders. Bioactive Food as Dietary Interventions for Arthritis and Related Inflammatory Diseases.

[25] Annadurai T., Thomas P.A., Geraldine P. (2013). Ameliorative Effect of Naringenin on Hyperglycemia-Mediated Inflammation in Hepatic and Pancreatic Tissues of Wistar Rats with Streptozotocin-Nicotinamide-Induced Experimental Diabetes Mellitus. Free Radic. Res..

[26] Kumar R., Tiku A.B. (2020). Naringenin Suppresses Chemically Induced Skin Cancer in Two-Stage Skin Carcinogenesis Mouse Model. Nutr. Cancer.

[27] Md S., Alhakamy N.A., Aldawsari H.M., Husain M., Kotta S., Abdullah S.T., Fahmy U.A., AlFaleh M.A., Asfour H.Z. (2020). Formulation Design, Statistical Optimization, and In Vitro Evaluation of a Naringenin Nanoemulsion to Enhance Apoptotic Activity in A549 Lung Cancer Cells. Pharmaceutics.

[28] Noori S., Rezaei Tavirani M., Deravi N., Mahboobi Rabbani M.I., Zarghi A. (2020). Naringenin Enhances the Anti-Cancer Effect of Cyclophosphamide against MDA-MB-231 Breast Cancer Cells Via Targeting the STAT3 Signaling Pathway. Iran. J. Pharm. Res..

[29] Zhao Z., Jin G., Ge Y., Guo Z. (2019). Naringenin Inhibits Migration of Breast Cancer Cells via Inflammatory and Apoptosis Cell Signaling Pathways. Inflammopharmacology.

[30] Lee J., Kim D.-H., Kim J.H. (2019). Combined Administration of Naringenin and Hesperetin with Optimal Ratio Maximizes the Anti-cancer Effect in Human Pan-Creatic Cancer via down Regulation of FAK and p38 Signaling Pathway. Phytomedicine.

[31] Han K.-Y., Chen P.-N., Hong M.-C., Hseu Y.-C., Chen K.-M., Hsu L.-S., Chen W.-J. (2018). Naringenin Attenuated Prostate Cancer Invasion via Reversal of Epithelial–to–Mesenchymal Transition and Inhibited uPA Activity. Anticancer Res..

[32] Lim W., Park S., Bazer F.W., Song G. (2017). Naringenin-Induced Apoptotic Cell Death in Prostate Cancer Cells Is Mediated via the PI3K/AKT and MAPK Signaling Pathways. J. Cell. Biochem..

[33] Bao L., Liu F., Guo H.-B., Li Y., Tan B.-B., Zhang W.-X., Peng Y.-H. (2016). Naringenin Inhibits Proliferation, Migration, and Invasion as Well as Induces Apoptosis of Gastric Cancer SGC7901 Cell Line by Downregulation of AKT Pathway. Tumor Biol..

[34] Ren B., Qin W., Wu F., Wang S., Pan C., Wang L., Zeng B., Ma S., Liang J. (2016). Apigenin and Naringenin Regulate Glucose and Lipid Metabolism, and Ameliorate Vascular Dysfunction in Type 2 Diabetic Rats. Eur. J. Pharmacol..

[35] Karim N., Jia Z., Zheng X., Cui S., Chen W. (2018). A Recent Review of Citrus Flavanone Naringenin on Metabolic Diseases and Its Potential Sources for High Yield-Production. Trends Food Sci. Technol..

[36] Yoshida H., Takamura N., Shuto T., Ogata K., Tokunaga J., Kawai K., Kai H. (2010). The Citrus Flavonoids Hesperetin and Naringenin Block the Lipolytic Actions of TNF-α in Mouse Adipocytes. Biochem. Biophys. Res. Commun..

[37] Chen S., Ding Y., Tao W., Zhang W., Liang T., Liu C. (2012). Naringenin Inhibits TNF-α Induced VSMC Proliferation and Migration via Induction of HO-1. Food Chem. Toxicol..

[38] Stacks N.M. (2015). Apigenin and Naringenin: Natural Sources, Pharmacology and Role in Cancer Prevention.

[39] Al-Rejaie S.S., Abuohashish H.M., Al-Enazi M.M., Al-Assaf A.H., Parmar M.Y., Ahmed M.M. (2013). Protective Effect of Naringenin on Acetic Acid-Induced Ulcerative Colitis in Rats. World J. Gastroenterol. WJG.

[40] Song H.-S., Bhatia S.K., Gurav R., Choi T.-R., Kim H.J., Park Y.-L., Han Y.-H., Park J.Y., Lee S.M., Park S.L. (2020). Naringenin as an Antibacterial Reagent Controlling of Biofilm Formation and Fatty Acid Metabolism in MRSA. bioRxiv.

[41] Martinez S.E., Lillico R., Lakowski T.M., Martinez S.A., Davies N.M. (2017). Pharmacokinetic Analysis of an Oral Multicomponent Joint Dietary Supplement (Phycox^®^) in Dogs. Pharmaceutics.

[42] Yang L.-J., Ma S.-X., Zhou S.-Y., Chen W., Yuan M.-W., Yin Y.-Q., Yang X.-D. (2013). Preparation and Characterization of Inclusion Complexes of Naringenin with β-Cyclodextrin or Its Derivative. Carbohydr. Polym..

[43] Shpigelman A., Shoham Y., Israeli-Lev G., Livney Y.D. (2014). β-Lactoglobulin–Naringenin Complexes: Nano-Vehicles for the Delivery of a Hydrophobic Nutraceutical. Food Hydrocoll..

[44] Kumar R.P., Abraham A. (2016). PVP-Coated Naringenin Nanoparticles for Biomedical Applications—In Vivo Toxicological Evaluations. Chem. Interact..

[45] Daeihamed M., Dadashzadeh S., Haeri A., Akhlaghi M.F. (2016). Potential of Liposomes for Enhancement of Oral Drug Absorption. Curr. Drug Deliv..

[46] Bayat F., Hosseinpour-Moghadam R., Mehryab F., Fatahi Y., Shakeri N., Dinarvand R., Hagen T.L.T., Haeri A. (2020). Potential Application of Liposomal Nanodevices for Non-Cancer Diseases: An Update on Design, Characterization and Biophar-Maceutical Evaluation. Adv. Colloid Interface Sci..

[47] Karuppusamy C., Venkatesan P. (2017). Role of Nanoparticles in Drug Delivery System: A Comprehensive Review. J. Pharm. Sci. Res..

[48] Zarepour A., Zarrabi A., Larsen K.L. (2019). Fabricating Β-Cyclodextrin Based Ph-Responsive Nanotheranostics as a Programmable Polymeric Nanocapsule for Simultaneous Diagnosis and Therapy. Int. J. Nanomed..

[49] Chakraborty K., Shivakumar A., Ramachandran S. (2016). Nano-Technology in Herbal Medicines: A Review. Int. J. Herb. Med..

[50] Dima C., Assadpour E., Dima S., Jafari S.M. (2020). Nutraceutical Nanodelivery; An Insight into the Bioaccessibility/Bioavailability of Different Bioactive Compounds Loaded within Nanocarriers. Crit. Rev. Food Sci. Nutr..

[51] Dima C., Assadpour E., Dima S., Jafari S.M. (2020). Bioactive-Loaded Nanocarriers for Functional Foods: From Designing to Bioavailability. Curr. Opin. Food Sci..

[52] Esfanjani A.F., Assadpour E., Jafari S.M. (2018). Improving the Bioavailability of Phenolic Compounds by Loading Them within Lipid-Based Nanocarriers. Trends Food Sci. Technol..

[53] Haeri A., Osouli M., Bayat F., Alavi S., Dadashzadeh S. (2017). Nanomedicine Approaches for Sirolimus Delivery: A Review of Pharmaceutical Properties and Preclinical Studies. Artif. Cells Nanomed. Biotechnol..

[54] Babadi D., Dadashzadeh S., Osouli M., Daryabari M.S., Haeri A. (2020). Nanoformulation Strategies for Improving Intestinal Permeability of Drugs: A More Precise Look at Permeability Assessment Methods and Pharmacokinetic Properties Changes. J. Control. Release.

[55] Detsi A., Kavetsou E., Kostopoulou I., Pitterou I., Pontillo A.R.N., Tzani A., Christodoulou P., Siliachli A., Zoumpoulakis P. (2020). Nanosystems for the Encapsulation of Natural Products: The Case of Chitosan Biopolymer as a Matrix. Pharmaceutics.

[56] Rahaiee S., Assadpour E., Esfanjani A.F., Silva A.S., Jafari S.M. (2020). Application of Nano/Microencapsulated Phenolic Compounds against Cancer. Adv. Colloid Interface Sci..

[57] Qin S.-Y., Zhang A.-Q., Cheng S.-X., Rong L., Zhang X.-Z. (2017). Drug Self-Delivery Systems for Cancer Therapy. Biomaterials.

[58] Akhlaghi S., Rabbani S., Alavi S., Alinaghi A., Radfar F., Dadashzadeh S., Haeri A. (2019). Green Formulation of Curcumin Loaded Lipid-Based Nanoparticles as a Novel Carrier for Inhibition of Post-Angioplasty Restenosis. Mater. Sci. Eng. C.

[59] Mohanty S., Sahoo A.K., Konkimalla V.B., Pal A., Si S.C. (2020). Naringin in Combination with Isothiocyanates as Liposomal Formulations Potentiates the Anti-inflammatory Activity in Different Acute and Chronic Animal Models of Rheumatoid Arthritis. ACS Omega.

[60] Fan H., Zhang P., Zhou L., Mo F., Jin Z., Ma J., Lin R., Liu Y., Zhang J. (2020). Naringin-Loaded Polymeric Micelles as Buccal Tablets: Formulation, Characterization, in Vitro Release, Cytotoxicity and Histo-Pathology Studies. Pharm. Dev. Technol..

[61] Zhu J., Huang Y., Zhang J., Feng Y., Shen L. (2020). Formulation, Preparation and Evaluation of Nanostructured Lipid Carrier Containing Naringin and Coix Seed Oil for Anti-Tumor Application Based on “Unification of Medicines and Excipients”. Drug Des. Dev. Ther..

[62] Mohamed E.A., Abu Hashim I.I., Yusif R.M., Shaaban A.A.A., El-Sheakh A.R., Hamed M.F., Badria F.A.E. (2018). Polymeric Micelles for Potentiated Antiulcer and Anticancer Activities of Naringin. Int. J. Nanomed..

[63] Assadpour E., Jafari S.M. (2020). Formulation and Application of Nanoemulsions for Nutraceuticals and Phytochemicals. Curr. Med. Chem..

[64] Maqsoudlou A., Assadpour E., Mohebodini H., Jafari S.M. (2020). Improving the Efficiency of Natural Antioxidant Compounds via Different Nanocarriers. Adv. Colloid Interface Sci..

[65] Hosseini H., Jafari S.M. (2020). Introducing Nano/Microencapsulated Bioactive Ingredients for Extending the Shelf-Life of Food Products. Adv. Colloid Interface Sci..

[66] Carissimi G., Montalbán M.G., Víllora G., Barth A. (2020). Direct Quantification of Drug Loading Content in Polymeric Nanoparticles by Infrared Spectroscopy. Pharmacy.

[67] Gera S., Talluri S., Rangaraj N., Sampathi S. (2017). Formulation and Evaluation of Naringenin Nanosuspensions for Bioavailability Enhancement. AAPS PharmSciTech.

[68] Akrawi S.H., Gorain B., Nair A.B., Choudhury H., Pandey M., Shah J.N., Venugopala K.N. (2020). Development and Optimization of Naringenin-Loaded Chitosan-Coated Nanoemulsion for Topical Therapy in Wound Healing. Pharmacy.

[69] Ghazy O., Fouad M., Saleh H., Kholif A., Morsy T. (2021). Ultrasound-Assisted Preparation of Anise Extract Nanoemulsion and Its Bioactivity against Different Pathogenic Bacteria. Food Chem..

[70] George D., Maheswari P.U., Begum K.M.S. (2020). Chitosan-Cellulose Hydrogel Conjugated with L-Histidine and Zinc Oxide Nanoparticles for Sustained Drug Delivery: Kinetics and in-Vitro Biological Studies. Carbohydr. Polym..

[71] Wang J., Ding Y., Zhou W. (2020). Albumin Self-Modified Liposomes for Hepatic Fibrosis Therapy via SPARC-Dependent Pathways. Int. J. Pharm..

[72] Hosny K.M., Alharbi W.S., Almehmady A.M., Bakhaidar R.B., Alkhalidi H.M., Sindi A.M., Hariri A.H., Shadab M., Zaki R.M. (2020). Preparation and Optimization of Pravastatin-Naringenin Nanotransfersomes to Enhance Bioavailability and Reduce Hepatic Side Effects. J. Drug Deliv. Sci. Technol..

[73] Yang F., Hu S., Sheng X., Liu Y. (2020). Naringenin Loaded Multifunctional Nanoparticles to Enhance the Chemotherapeutic Efficacy in Hepatic Fibrosis. Biomed. Microdevices.

[74] Padmanabhan V.P., Balakrishnan S., Kulandaivelu R., Narayanan S.N.T.S., Lakshmipathy M., Sagadevan S., Mohammad F., Al-Lohedan H.A., Paiman S., Oh W.C. (2020). Nanoformulations of Core–Shell Type Hydroxyapatite-Coated Gum Acacia with Enhanced Bioactivity and Controlled Drug De-livery for Biomedical Applications. New J. Chem..

[75] Yu Z., Liu X., Chen H., Zhu L. (2020). Naringenin-Loaded Dipalmitoylphosphatidylcholine Phytosome Dry Powders for Inhaled Treatment of Acute Lung Injury. J. Aerosol Med. Pulm. Drug Deliv..

[76] Guan M., Shi R., Zheng Y., Zeng X., Fan W., Wang Y., Su W. (2020). Characterization, in Vitro and in Vivo Evaluation of Naringenin-Hydroxypropyl-β-Cyclodextrin Inclusion for Pulmonary Delivery. Molecules.

[77] Fuster M.G., Carissimi G., Montalbán M.G., Víllora G. (2020). Improving Anticancer Therapy with Naringenin-Loaded Silk Fibroin Nanoparticles. Nanomaterials.

[78] Tsai M.-J., Huang Y.-B., Fang J.-W., Fu Y.-S., Wu P.-C. (2015). Preparation and Characterization of Naringenin-Loaded Elastic Liposomes for Topical Application. PLoS ONE.

[79] Krishnakumar N., Sulfikkarali N., Rajendraprasad N., Karthikeyan S. (2011). Enhanced Anticancer Activity of Naringenin-Loaded Nanoparticles in Human Cervical (HeLa) Cancer Cells. Biomed. Prev. Nutr..

[80] Wu C., Ji P., Yu T., Liu Y., Jiang J., Xu J., Zhao Y., Hao Y., Qiu Y., Zhao W. (2016). Naringenin-Loaded Solid Lipid Nanoparticles: Preparation, Controlled Delivery, Cellular Uptake, and Pulmonary Pharmacokinetics. Drug Des. Dev. Ther..

[81] Shadab A.N.A., Aldawsari H.M., Asfour H.Z. (2019). Neuroprotective and Antioxidant Effect of Naringenin-Loaded Nanoparticles for Nose-to-Brain Delivery. Brain Sci..

[82] Wang H., Li X., Yang H., Wang J., Li Q., Qu R., Wu X. (2020). Nanocomplexes Based Polyvinylpyrrolidone K-17PF for Ocular Drug Delivery of Naringenin. Int. J. Pharm..

[83] Zhang P., Liu X., Hu W., Bai Y., Zhang L. (2016). Preparation and Evaluation of Naringenin-Loaded Sulfobutylether-β-Cyclodextrin/Chitosan Nanoparticles for Ocular Drug Delivery. Carbohydr. Polym..

[84] Morais R.P., Novais G.B., Sangenito L.S., Santos A.L.S., Priefer R., Morsink M., Mendonça M.C., Souto E.B., Severino P., Cardoso J.C. (2020). Naringenin-Functionalized Multi-Walled Carbon Nanotubes: A Potential Approach for Site-Specific Remote-Controlled Anticancer Delivery for the Treatment of Lung Cancer Cells. Int. J. Mol. Sci..

[85] Kanaze I.F., Bounartzi I.M., Georgarakis M., Niopas I. (2006). Pharmacokinetics of the Citrus Flavanone Aglycones Hesperetin and Naringenin after Single Oral Administration in Human Subjects. Eur. J. Clin. Nutr..

[86] Najmanova I., Vopršalová M., Saso L., Mladěnka P. (2020). The Pharmacokinetics of Flavanones. Crit. Rev. Food Sci. Nutr..

[87] Bai Y., Peng W., Yang C., Zou W., Liu M., Wu H., Fan L., Li P., Zeng X., Su W. (2020). Pharmacokinetics and Metabolism of Naringin and Active Metabolite Naringenin in Rats, Dogs, Humans, and the Differences Between Species. Front. Pharmacol..

[88] Pimpão R.C., Ventura M.R., Ferreira R.B., Williamson G., Santos C.N. (2015). Phenolic Sulfates as New and Highly Abundant Metabolites in Human Plasma after Ingestion of a Mixed Berry Fruit Purée. Br. J. Nutr..

[89] Feliciano R.P., Boeres A., Massacessi L., Istas G., Ventura M.R., Dos Santos C.N., Heiss C., Rodriguez-Mateos A. (2016). Identification and Quantification of Novel Cranberry-Derived Plasma and Urinary (Poly)Phenols. Arch. Biochem. Biophys..

[90] Venditti I. (2019). Morphologies and Functionalities of Polymeric Nanocarriers as Chemical Tools for Drug Delivery: A Review. J. King Saud Univ. Sci..

[91] Amoabediny G., Haghiralsadat F., Naderinezhad S., Helder M.N., Kharanaghi E.A., Arough J.M., Zandieh-Doulabi B. (2017). Overview of Preparation Methods of Polymeric and Lipid-Based (Niosome, Solid Lipid, Liposome) Nanoparticles: A Comprehensive Review. Int. J. Polym. Mater..

[92] George A., Shah P.A., Shrivastav P.S. (2019). Natural Biodegradable Polymers Based Nano-Formulations for Drug Delivery: A Review. Int. J. Pharm..

[93] Jahangiri A., Barghi L. (2018). Polymeric Nanoparticles: Review of Synthesis Methods and Applications in Drug Delivery. J. Adv. Chem. Pharm. Mater..

[94] Rehman A., Jafari S.M., Aadil R.M., Assadpour E., Randhawa M.A., Mahmood S. (2020). Development of Active Food Packaging via Incorporation of Biopolymeric Nanocarriers Containing Essential Oils. Trends Food Sci. Technol..

[95] Rostami M.R., Yousefi M., Khezerlou A., Mohammadi M.A., Jafari S.M. (2019). Application of Different Biopolymers for Nanoencapsulation of Antioxidants via Electrohydrodynamic Processes. Food Hydrocoll..

[96] Akbari-Alavijeh S., Shaddel R., Jafari S.M. (2020). Encapsulation of Food Bioactives and Nutraceuticals by Various Chitosan-Based Nanocarriers. Food Hydrocoll..

[97] Yousefi M., Narmani A., Jafari S.M. (2020). Dendrimers as Efficient Nanocarriers for the Protection and Delivery of Bioactive Phytochemicals. Adv. Colloid Interface Sci..

[98] Song I.-S., Cha J.-S., Choi M.-K. (2015). Enhanced Oral Bioavailability of Naringenin Administered in a Mixed Micelle Formulation with Pluronic F127 and Tween 80 in Rats. J. Pharm. Investig..

[99] Domínguez-Delgado C.L., Fuentes-Prado E., Escobar-Chávez J.J., Vidal-Romero G., Rodríguez Cruz I., Díaz-Torres R. (2016). Chitosan and Pluronic^®^ F-127: Pharmaceutical Applications. Encyclopedia of Biomedical Polymers and Polymeric Biomaterials.

[100] Cheng M., Zeng G., Huang D., Yang C., Lai C., Zhang C., Liu Y. (2017). Advantages and Challenges of Tween 80 Surfactant-Enhanced Technologies for the Remediation of Soils Contaminated with Hydrophobic Organic Compounds. Chem. Eng. J..

[101] Li M., Wang K., Wang Y., Han Q., Ni Y., Wen X. (2020). Effects of Genipin Concentration on Cross-Linked β-Casein Micelles as Nanocarrier of Naringenin: Colloidal Properties, Structural Characterization and Controlled Release. Food Hydrocoll..

[102] Chen H., Wooten H., Thompson L., Pan K. (2019). Nanoparticles of Casein Micelles for Encapsulation of Food Ingredients. Biopolymer Nanostructures for Food Encapsulation Purposes.

[103] Moeiniafshari A.-A., Zarrabi A., Bordbar A.-K. (2015). Exploring the Interaction of Naringenin with Bovine Beta-Casein Nanoparticles Using Spectroscopy. Food Hydrocoll..

[104] Yin X., Fu X., Cheng H., Wusigale L.L. (2020). α-Tocopherol and Naringenin in Whey Protein Isolate Particles: Partition, Antioxidant Activity, Stability and Bioaccessibility. Food Hydrocoll..

[105] Wang K., Liu T., Lin R., Liu B., Yang G., Bu X., Wang W., Zhang P., Zhou L., Zhang J. (2014). Preparation and in Vitro Release of Buccal Tablets of Naringenin-Loaded MPEG-PCL Nanoparticles. RSC Adv..

[106] Muralidharan S., Shanmugam K. (2020). Synthesis and Characterization of Naringenin-Loaded Chitosan-Dextran Sulfate Nanocarrier. J. Pharm. Innov..

[107] Gordillo-Galeano A., Mora-Huertas C.E. (2018). Solid Lipid Nanoparticles and Nanostructured Lipid Carriers: A Review Emphasizing on Particle Structure and Drug Release. Eur. J. Pharm. Biopharm..

[108] Mohammadi-Samani S., Ghasemiyeh P. (2018). Solid Lipid Nanoparticles and Nanostructured Lipid Carriers as Novel Drug Delivery Systems: Applications, Advantages and Disadvantages. Res. Pharm. Sci..

[109] Rostamabadi H., Falsafi S.R., Jafari S.M. (2019). Nanoencapsulation of Carotenoids within Lipid-Based Nanocarriers. J. Control. Release.

[110] Mohammadi M., Assadpour E., Jafari S.M. (2019). Encapsulation of Food Ingredients by Nanostructured Lipid Carriers (NLCs). Lipid-Based Nanostructures for Food Encapsulation Purposes.

[111] Akhavan S., Assadpour E., Katouzian I., Jafari S.M. (2018). Lipid Nano Scale Cargos for the Protection and Delivery of Food Bioactive Ingredients and Nutraceuticals. Trends Food Sci. Technol..

[112] Katouzian I., Esfanjani A.F., Jafari S.M., Akhavan S. (2017). Formulation and Application of a New Generation of Lipid Nano-Carriers for the Food Bioactive Ingredients. Trends Food Sci. Technol..

[113] Wang Y., Wang S., Firempong C.K., Zhang H., Wang M., Zhang Y., Zhu Y., Yuanwen W., Xu X. (2017). Enhanced Solubility and Bioavailability of Naringenin via Liposomal Nanoformulation: Preparation and In Vitro and In Vivo Evaluations. AAPS Pharmscitech.

[114] Raeisi S., Chavoshi H., Mohammadi M., Ghorbani M., Sabzichi M., Ramezani F. (2019). Naringenin-Loaded Nano-Structured Lipid Carrier Fortifies Oxaliplatin-Dependent Apoptosis in HT-29 Cell Line. Process. Biochem..

[115] Zhu Y., Wang M., Zhang J., Peng W., Firempong C.K., Deng W., Wang Q., Wang S., Shi F., Yu J. (2015). Improved Oral Bioavailability of Capsaicin via Liposomal Nanoformulation: Preparation, in Vitro Drug Release and PharmaCo-Kinetics in Rats. Arch. Pharmacol. Res..

[116] Haghiralsadat F., Amoabediny G., Sheikhha M.H., Zandieh-doulabi B., Naderinezhad S., Helder M.N., Forouzanfar T. (2017). New Liposomal Doxorubicin Nanoformulation for Osteosarcoma: Drug Release Kinetic Study Based on Thermo and pH Sensitivity. Chem. Biol. Drug Des..

[117] Jain A.K., Swarnakar N.K., Godugu C., Singh R.P., Jain S. (2011). The Effect of the Oral Administration of Polymeric Nanoparticles on the Efficacy and Toxicity of Tamoxifen. Biomaterials.

[118] Karadag A., Ozcelik B., Huang Q. (2014). Quercetin Nanosuspensions Produced by High-Pressure Homogenization. J. Agric. Food Chem..

[119] Mauludin R., Müller R.H. (2013). Preparation and Storage Stability of Rutin Nanosuspensions. J. Pharm. Investig..

[120] Jacob S., Nair A.B., Shah J. (2020). Emerging Role of Nanosuspensions in Drug Delivery Systems. Biomater. Res..

[121] Singh M.K., Pooja D., Ravuri H.G., Gunukula A., Kulhari H., Sistla R. (2018). Fabrication of Surfactant-Stabilized Nanosuspension of Naringenin to Surpass Its Poor Physiochemical Properties and Low Oral Bioavailability. Phytomedicine.

[122] Rajamani S., Radhakrishnan A., Sengodan T., Thangavelu S. (2018). Augmented Anticancer Activity of Naringenin-Loaded TPGS Polymeric Nanosuspension for Drug Resistive MCF-7 Human Breast Cancer Cells. Drug Dev. Ind. Pharm..

[123] Zhao S., Yang X., Garamus V.M., Handge U.A., Bérengère L., Zhao L., Salamon G., Willumeit R., Zou A., Fan S. (2014). Mixture of Nonionic/Ionic Surfactants for the Formulation of Nanostructured Lipid Carriers: Effects on Physical Properties. Langmuir.

[124] Rajamani S., Kalyanasundaram G., Sengodan T., Thangavelu S., Shanmukhan N.K., Radhakrishnan A. (2019). Hepato & Nephro Protective Effects of Naringenin-Loaded Tpgs Polymeric Nanosuspension against Cisplatin-Induced Toxicity. Int. J. Res. Pharm. Sci..

[125] Sumathi R., Tamizharasi S., Sivakumar T. (2017). Formulation and Evaluation of Polymeric Nanosuspension of Naringenin. Int. J. Appl. Pharm..

[126] Khan A.W., Kotta S., Ansari S.H., Sharma R.K., Ali J. (2015). Self-Nanoemulsifying Drug Delivery System (SNEDDS) of the Poorly Water-Soluble Grapefruit Flavonoid Naringenin: Design, Characterization, in Vitroandin Vivoevaluation. Drug Deliv..

[127] Fuior E.V., Mocanu C.A., Deleanu M., Voicu G., Anghelache M., Rebleanu D., Simionescu M., Calin M. (2020). Evaluation of VCAM-1 Targeted Naringenin/Indocyanine Green-Loaded Lipid Nanoemulsions as Theranostic Nanoplatforms in Inflammation. Pharm..

[128] Fuior E.V., Deleanu M., Constantinescu C.A., Rebleanu D., Voicu G., Simionescu M., Calin M. (2019). Functional Role of VCAM-1 Targeted Flavonoid-Loaded Lipid Nanoemulsions in Reducing Endothelium Inflammation. Pharmaceutics.

[129] Kanaze F., Kokkalou E., Niopas I., Barmpalexis P., Georgarakis E., Bikiaris D. (2010). Dissolution Rate and Stability Study of Flavanone Aglycones, Naringenin and Hesperetin, by Drug Delivery Systems Based on Polyvinylpyrrolidone (PVP) Nanodispersions. Drug Dev. Ind. Pharm..

[130] Kerdudo A., Dingas A., Fernandez X., Faure C. (2014). Encapsulation of Rutin and Naringenin in Multilamellar Vesicles for Optimum Antioxidant Activity. Food Chem..

[131] Sandhu P.S., Kumar R., Beg S., Jain S., Kushwah V., Katare O., Singh B. (2017). Natural Lipids Enriched Self-Nano-Emulsifying Systems for Effective Co-Delivery of Tamoxifen and Naringenin: Systematic AP-Proach for Improved Breast Cancer Therapeutics. Nanomed. Nanotechnol. Biol. Med..

[132] Ahmad M.Z., Ahmad J., Zafar S., Warsi M.H., Abdel-Wahab B.A., Akhter S., Alam A. (2020). Omega-3 Fatty Acids as Adjunctive Therapeutics: Prospective of Nanoparticles in Its Formulation Development. Ther. Deliv..

[133] Dasgupta S., Bhattacharyya D.K. (2007). Dietary Effect of Eicosapentaenoic Acid (EPA) Containing Soyphospholipid. J. Oleo Sci..

[134] Shirouchi B., Nagao K., Inoue N., Ohkubo T., Hibino A.H., Yanagita T. (2007). Effect of Dietary Omega 3 Phosphatidylcholine on Obesity-Related Disorders in Obese Otsuka Long-Evans Tokushima Fatty Rats. J. Agric. Food Chem..

[135] Parashar P., Rathor M., Dwivedi M., Saraf S.A. (2018). Hyaluronic Acid Decorated Naringenin Nanoparticles: Appraisal of Chemopreventive and Curative Potential for Lung Cancer. Pharmaceutics.

[136] Chaurasia S., Patel R.R., Vure P., Mishra B. (2018). Potential of Cationic-Polymeric Nanoparticles for Oral Delivery of Naringenin: In Vitro and In Vivo Investigations. J. Pharm. Sci..

[137] Akhter H., Kumar S., Nomani S. (2020). Sonication Tailored Enhance Cytotoxicity of Naringenin Nanoparticle in Pancreatic Cancer: Design, Optimization, and in Vitro Studies. Drug Dev. Ind. Pharm..

[138] Gaba B., Khan T., Haider F., Alam T., Baboota S., Parvez S., Ali J. (2019). Vitamin E Loaded Naringenin Nanoemulsion via Intranasal Delivery for the Management of Oxidative Stress in a 6-OHDA Parkinson’s Disease Model. BioMed Res. Int..

[139] Abraham R.P.K.A. (2017). Inhibition of LPS Induced Pro-Inflammatory Responses in RAW 264.7 Macrophage Cells by PVP-Coated Naringenin Nanoparticle via down Regulation of NF-κB/P38MAPK Mediated Stress Signaling. Pharmacol. Rep..

[140] Chen C., Jie X., Ou Y., Cao Y., Xu L., Wang Y., Qi R. (2017). Nanoliposome Improves Inhibitory Effects of Naringenin on Nonalcoholic Fatty Liver Disease in Mice. Nanomedicine.

[141] Maity S., Chakraborti A.S. (2020). Formulation, PhysiCo-chemical Characterization and Antidiabetic Potential of Naringenin-Loaded Poly D, L Lactide-Co-Glycolide (N-PLGA) Nanoparticles. Eur. Polym. J..

[142] George D., Maheswari P.U., Begum K.M.S. (2020). Cysteine Conjugated Chitosan Based Green Nanohybrid Hydrogel Embedded with Zinc Oxide Nanoparticles Towards Enhanced Therapeutic Potential of Naringenin. React. Funct. Polym..

[143] Zhang H., Zhong X., Zhang X., Shang D., Zhou Y., Zhang C. (2015). Enhanced Anticancer Effect of ABT-737 in Combination with Naringenin on Gastric Cancer Cells. Exp. Ther. Med..

[144] Chang H.-L., Chang Y.-M., Lai S.-C., Chen K.-M., Wang K.-C., Chiu T.-T., Chang F.-H., Hsu L.-S. (2017). Naringenin Inhibits Migration of Lung Cancer Cells via the Inhibition of Matrix Metalloproteinases-2 and-9. Exp. Ther. Med..

[145] Mir I.A., Tiku A.B. (2014). Chemopreventive and Therapeutic Potential of “Naringenin,” a Flavanone Present in Citrus Fruits. Nutr. Cancer.

[146] Arafah A., Rehman M.U., Mir T.M., Wali A.F., Ali R., Qamar W., Khan R., Ahmad A., Aga S.S., Alqahtani S. (2020). Multi-Therapeutic Potential of Naringenin (4′,5,7-Trihydroxyflavonone): Experimental Evidence and Mechanisms. Plants.

[147] Chaurasia S., Patel R.R., Vure P., Mishra B. (2017). Oral Naringenin Nanocarriers: Fabrication, Optimization, Pharmacokinetic and Chemotherapeutic Efficacy Assessments. Nanomedicine.

[148] Kumar S.P., Birundha K., Kaveri K., Devi K.R. (2015). Antioxidant Studies of Chitosan Nanoparticles Containing Naringenin and Their Cytotoxicity Effects in Lung Cancer Cells. Int. J. Biol. Macromol..

[149] Krishnakumar N., Sulfikkarali N.K., Manoharan S., Nirmal R.M. (2013). Screening of Chemopreventive Effect of Naringenin-Loaded Nanoparticles in DMBA-Induced Hamster Buccal Pouch Carcino-Genesis by FT-IR Spectroscopy. Mol. Cell. Biochem..

[150] Sulfikkarali N., Krishnakumar N., Manoharan S., Nirmal R.M. (2012). Chemopreventive Efficacy of Naringenin-Loaded Nanoparticles in 7,12-Dimethylbenz(a)anthracene Induced Experimental Oral Carcinogenesis. Pathol. Oncol. Res..

[151] Krishnakumar N., Sulfikkarali N., Manoharan S., Venkatachalam P. (2013). Raman Spectroscopic Investigation of the Chemopreventive Response of Naringenin and Its Nanoparticles in DMBA-Induced Oral Carcinogenesis. Spectrochim. Acta Part A Mol. Biomol. Spectrosc..

[152] Sulfikkarali N.K., Krishnakumar N. (2013). Evaluation of the Chemopreventive Response of Naringenin-Loaded Nanoparticles in Experimental Oral Carcinogenesis Using Laser-Induced Autofluorescence Spectroscopy. Laser Phys..

[153] Feigin V.L., Nichols E., Alam T., Bannick M.S., Beghi E., Blake N., Culpepper W.J., Dorsey E.R., Elbaz A., Ellenbogen R.G. (2019). Global, Regional, and National Burden of Neurological Disorders, 1990–2016: A Systematic Analysis for the Global Burden of Disease Study 2016. Lancet Neurol..

[154] Zhou Y., Peng Z., Seven E.S., Leblanc R.M. (2018). Crossing the Blood-Brain Barrier with Nanoparticles. J. Control. Release.

[155] Ahmad N., Ahmad R., Ahmad F.J., Ahmad W., Alam A., Amir M., Ali A. (2020). Poloxamer-Chitosan-Based Naringenin Nanoformulation Used in Brain Targeting for the Treatment of Cerebral Ischemia. Saudi J. Biol. Sci..

[156] Md S., Gan S.Y., Haw Y.H., Ho C.L., Wong S., Choudhury H. (2018). In Vitro Neuroprotective Effects of Naringenin Nanoemulsion against β-Amyloid Toxicity through the Regulation of Amyloido-Genesis and Tau Phosphorylation. Int. J. Biol. Macromol..

[157] Salehi B., Rescigno A., Dettori T., Calina D., Docea A.O., Singh L., Cebeci F., Özçelik B., Bhia M., Beirami A.D. (2020). Avocado–Soybean Unsaponifiables: A Panoply of Potentialities to Be Exploited. Biomolecules.

[158] Kunnumakkara A.B., Sailo B.L., Banik K., Harsha C., Prasad S., Gupta S.C., Bharti A.C., Aggarwal B.B. (2018). Chronic Diseases, Inflammation, and Spices: How Are They Linked?. J. Transl. Med..

[159] Roy N.K., Parama D., Banik K., Bordoloi D., Devi A.K., Thakur K.K., Padmavathi G., Shakibaei M., Fan L., Sethi G. (2019). An Update on Pharmacological Potential of Boswellic Acids against Chronic Diseases. Int. J. Mol. Sci..

[160] TuTunchi H., Naeini F., Ostadrahimi A., Hosseinzadeh-Attar M.J. (2020). Naringenin, a Flavanone with Antiviral and Anti-Inflammatory Effects: A Promising Treatment Strategy Against COVID-19. Phytother. Res..

[161] Ahmad A., Fauzia E., Kumar M., Mishra R.K., Kumar A., Khan M.A., Raza S.S., Khan R. (2018). Gelatin-Coated Polycaprolactone Nanoparticle-Mediated Naringenin Delivery Rescue Human Mesenchymal Stem Cells from Oxygen Glucose Deprivation-Induced Inflammatory Stress. ACS Biomater. Sci. Eng..

[162] Mulyani H., Dewi R.T., Chaidir C. (2019). ANTIBACTERIAL COMPOUND of Aspergillus Elegans SWEF9 Endophytic Fungi from Seaweed. Indones. J. Pharm..

[163] Kim T.-H., Kim G.-D., Ahn H.-J., Cho J.-J., Park Y.S., Park C.-S. (2013). The Inhibitory Effect of Naringenin on Atopic Dermatitis Induced by DNFB in NC/Nga Mice. Life Sci..

[164] Gaggeri R., Rossi D., Hajikarimian N., Martino E., Bracco F., Grisoli P., Dacarro C., Leoni F., Mascheroni G., Collina S. (2010). Preliminary Study on TNFα-Blocker Activity of Amygdalus lycioides Spach Extracts. Open Nat. Prod. J..

[165] Gaggeri R., Rossi D., Christodoulou M.S., Passarella D., Leoni F., Azzolina O., Collina S. (2012). Chiral Flavanones from Amygdalus Lycioides Spach: Structural Elucidation and Identification of TNFalpha Inhibitors by Bioactivity-guided Fractionation. Molecules.

[166] Chlapanidas T., Perteghella S., Leoni F., Faragò S., Marazzi M., Rossi D., Martino E., Gaggeri R., Collina S. (2014). TNF-α Blocker Effect of Naringenin-Loaded Sericin Microparticles that Are Potentially Useful in the Treatment of Psoriasis. Int. J. Mol. Sci..

[167] Tsai M.-J., Huang Y.-B., Fang J.-W., Fu Y.-S., Jhih-Wun F. (2015). Preparation and Evaluation of Submicron-Carriers for Naringenin Topical Application. Int. J. Pharm..

[168] Carreras J.J., Ramirez E.T.W., Sala A., Guillot A.J., Garrigues T.M., Melero A. (2019). Ultraflexible Lipid Vesicles Allow Topical Absorption of Cyclosporin A. Drug Deliv. Transl. Res..

[169] Castoldi A., Herr C., Niederstraßer J., Labouta H.I., Melero A., Gordon S., Schneider-Daum N., Bals R., Lehr C.-M. (2017). Calcifediol-Loaded Liposomes for Local Treatment of Pulmonary Bacterial Infections. Eur. J. Pharm. Biopharm..

[170] Dreier J., Sørensen J.A., Brewer J.R. (2016). Superresolution and Fluorescence Dynamics Evidence Reveal That Intact Liposomes Do Not Cross the Human Skin Barrier. PLoS ONE.

[171] Martinez R.M., Pinho-Ribeiro F.A., Steffen V.S., Caviglione C.V., Vignoli J.A., Barbosa D.S., Baracat M.M., Georgetti S.R., Verri W.A., Casagrande R. (2015). Naringenin Inhibits UVB Irradiation-Induced Inflammation and Oxidative Stress in the Skin of Hairless Mice. J. Nat. Prod..

[172] Martinez R.M., Pinho-Ribeiro F.A., Steffen V.S., Silva T.C.C., Caviglione C.V., Bottura C., Fonseca M.J.V., Vicentini F.T.M.C., Vignoli J.A., Baracat M.M. (2016). Topical Formulation Containing Naringenin: Efficacy against Ultraviolet B Irradiation-Induced Skin Inflammation and Oxidative Stress in Mice. PLoS ONE.

[173] Badea G., Badea N., Brasoveanu L.I., Mihaila M., Stan R., Istrati D., Balaci T., Lacatusu I. (2016). Naringenin Improves the Sunscreen Performance of Vegetable Nanocarriers. New J. Chem..

[174] Joshi H., Hegde A.R., Shetty P.K., Gollavilli H., Managuli R.S., Kalthur G., Mutalik S. (2018). Sunscreen Creams Containing Naringenin Nanoparticles: Formulation Development and in Vitro and in Vivo Evaluations. Photodermatol. Photoimmunol. Photomed..

[175] Al-Dosari D.I., Ahmed M.M., Al-Rejaie S.S., Alhomida A.S., Ola M.S. (2017). Flavonoid Naringenin Attenuates Oxidative Stress, Apoptosis and Improves Neurotrophic Effects in the Diabetic Rat Retina. Nutrients.

[176] Oguido A.P.M.T., Hohmann M.S.N., Pinho-Ribeiro F.A., Crespigio J., Domiciano T.P., Verri W.A., Casella A.M.B. (2017). Naringenin Eye Drops Inhibit Corneal Neovascularization by Anti-Inflammatory and Antioxidant Mechanisms. Investig. Opthalmol. Vis. Sci..

[177] Shiratori K., Ohgami K., Ilieva I., Jin X.-H., Yoshida K., Kase S., Ohno S. (2005). The Effects of Naringin and Naringenin on Endotoxin-Induced Uveitis in Rats. J. Ocul. Pharmacol. Ther..

[178] Sahu N., Mishra G., Chandra H.K., Nirala S.K., Bhadauria M. (2020). Naringenin Mitigates Antituberculosis Drugs Induced Hepatic and Renal Injury in Rats. J. Tradit. Complement. Med..

[179] Jain A., Yadav A., Bozhkov A., Padalko V., Flora S. (2011). Therapeutic Efficacy of Silymarin and Naringenin in Reducing Arsenic-Induced Hepatic Damage in Young Rats. Ecotoxicol. Environ. Saf..

[180] Yen F.-L., Wu T.-H., Lin L.-T., Cham T.-M., Lin C.-C. (2008). Naringenin-Loaded Nanoparticles Improve the Physicochemical Properties and the Hepatoprotective Effects of Naringenin in Orally-Administered Rats with CCl4-Induced Acute Liver Failure. Pharm. Res..

[181] Maity S., Mukhopadhyay P., Kundu P.P., Chakraborti A.S. (2017). Alginate Coated Chitosan Core-Shell Nanoparticles for Efficient Oral Delivery of Naringenin in Diabetic Animals—an in Vitro and in Vivo Approach. Carbohydr. Polym..

